# Decisive gene strategy on osteoporosis: a comprehensive whole-literature-based approach for conclusive candidate gene targets

**DOI:** 10.18632/aging.204026

**Published:** 2022-04-22

**Authors:** Yueh-Chun Chen, Yu-Jui Tsai, Chih-Chien Wang, Pi-Shao Ko, Wen Su, Sui-Lung Su

**Affiliations:** 1 Graduate Institute of Medical Sciences, National Defense Medical Center, Taipei, Taiwan, R.O.C; 2 School of Public Health, National Defense Medical Center, Taipei, Taiwan, R.O.C; 3 Department of Orthopedics, Tri-Service General Hospital, National Defense Medical Center, Taipei, Taiwan, R.O.C; 4 Graduate Institute of Life Sciences, National Defense Medical Center, Taipei, Taiwan, R.O.C; 5 Graduate Institute of Aerospace and Undersea Medicine, National Defense Medical Center, Taipei, Taiwan, R.O.C

**Keywords:** osteoporosis, trial sequential analysis, polymorphism, meta-analysis

## Abstract

Purpose: Previous meta-analyses only examined the association between single gene polymorphisms and osteoporosis; there is no compilation of all gene loci that correlate with osteoporosis in the literature. In this study, we develop a new literature-based approach, a decisive gene strategy (DGS), to examine the sufficiency of the cumulative sample size for each gene locus and to assess whether a definite conclusion of the association between the gene locus and osteoporosis can be drawn.

Methods: The DGS was used to search PubMed, Embase, and Cochrane databases for all meta-analyses that correlated gene polymorphisms with osteoporosis. Trial sequential analysis was employed to examine the sufficiency of the cumulative sample size. Finally, we assessed the importance of gene loci in osteoporosis based on whether there were enough sample sizes and the heterogeneity of the literature with the I^2^ value.

Results: After excluding 169 irrelevant publications, 39 meta-analysis papers were obtained. Among Caucasians, in 17 gene loci, there were eight gene loci (e.g., vitamin D Receptor ApaI rs7975232) with sufficient cumulative sample size to confirm that they were unrelated to the disease. Among Asians, in 15 gene loci, four gene loci that had sufficient sample sizes were risk factors: VDR FokI rs2228570 (odds ratio (OR) = 1.44, 95% confidence interval (CI) = 1.22–1.70), TGF β1 rs1800470 (OR = 1.35, 95% CI = 1.10–1.65), IGF1 rs2288377 (OR = 1.44, 95% CI = 1.28–1.62), and IGF1 rs35767 (OR = 1.20, 95% CI = 1.06–1.36), respectively, whereas one gene locus, ESR2 RsaI rs1256049 (OR = 0.69, 95% CI = 0.59–0.81), was a protective factor.

Conclusions: The DGS successfully identified five gene loci in osteoporosis that will apply to other diseases to find causal genes, which may contribute to further genetic therapy.

## INTRODUCTION

In the genetic epidemiology field, enormous resources have been invested globally in gene studies. Genome-wide association studies (GWAS) are used to identify gene mutations and to assess their correlation with a disease. Numerous genetic research papers are published yearly. In these papers, in the search for trait-related biomarkers, approximately 200 single-nucleotide polymorphisms (SNPs) associated with common phenotypes have been discovered. However, despite the seemingly numerous SNPs, we found that these SNPs only explains a small proportion of variations in complex traits. This phenomenon is called the “missing heritability” problem [1]. For instance, previous studies have calculated that height heritability is 60%–80% [2]. Nevertheless, the most important 40 SNPs discovered previously can predict only 5% of the total height-related variations [3]. Such deficiencies are present in other complex traits too. Therefore, research on the missing heritability is currently the most crucial issue in human genetics, and in-depth investigation of conventional studies is still required [1].

Among conventional studies, meta-analysis provides the highest level of evidence. However, previous meta-analyses on gene polymorphisms and diseases have two difficulties: (1) most studies only examined a single gene or locus and do not identify or analyze all disease-related genes or loci; (2) it is impossible to determine whether the cumulative sample size for a gene/locus is sufficient, and no additional resources and samples are required for study [4]. Therefore, this study developed a decisive gene strategy (DGS) to resolve these two difficulties.

Many previous meta-analyses examined the correlation between gene polymorphisms and osteoporosis. After searching the PubMed, Embase, and Cochrane databases, we found 65, 183, and 5 meta-analyses, respectively that examined the correlation between gene polymorphisms and osteoporosis. We used the DGS for gene locus screening. A whole-literature-based approach was adopted to identify all osteoporosis-related gene loci, and their correlation with osteoporosis was summarized based on existing evidence.

## MATERIALS AND METHODS

### DGS

Keyword search and trial sequential analysis (TSA) approaches were used to develop the DGS. In this strategy, keyword search was first employed to search the literature, using SCI-indexed papers as the basis, for published meta-analyses that examined the correlation between gene polymorphisms and osteoporosis. Next, the gene distribution frequencies of various gene loci were extracted from the publications. Afterward, TSA was employed to examine the sufficiency of the cumulative sample sizes of the various gene loci for a conclusion, such as which gene loci were protective or risk factors for a disease and which gene loci were not associated with the disease. By employing these two approaches in the DGS, a complete current status of genes associated with osteoporosis can be constructed.

### Keyword search

The PubMed, Embase, and Cochrane databases were searched for meta-analyses that examined the correlation between gene polymorphisms and osteoporosis. The keywords used included synonyms of osteoporosis, gene polymorphism, and meta-analysis (see Supplementary Table 1 for details). After the keyword search, the first author, publication year, refSNP (rs) number, and number of subjects in the case and control groups and their respective genotype distributions were extracted. Additionally, the genotype-tissue expression (GTEx) database was employed to clarify the mRNA expression level of each gene loci. The eventual goal of the recently announced GTEx project is to create a whole-body map of haplotypic expression so that any risk haplotype for any disease can be easily checked for its effect on genome-wide and tissue-wide RNA expression [5]. We extracted the mRNA expression level of all SNPs and their downstream genes in blood to show the correlation between the SNPs and functions.

### TSA

The publication date of papers was used as a starting point. All new samples and previous cumulative samples included in the TSA were combined and analyzed to calculate the required information size (RIS) for TSA and to provide the monitoring and futility boundaries for hypothesis testing [6]. The statistical validation results of TSA tended to be stable only when the study’s cumulative sample size reached the RIS or when the Z curve in hypothesis testing intersected with the monitoring or futility boundary.

The principle of TSA is to consider meta-analysis as multiple tests, and one additional test is performed every time a new study sample is added. TSA can be employed to correct inflated P values caused by multiple tests and to decrease the type I error’s occurrence risk. Additionally, TSA formulates two curves for the cumulative sample size: the O’Brien–Fleming and invalid boundaries. The O’Brien–Fleming boundary is plotted according to the quantitation of random error and heterogeneity of accumulated papers, and the invalid boundary is based on a similar theory. Therefore, the two curves can ensure that significant differences are not due to the study results’ excessive inflation.

### Statistical analysis

As the minor allele frequency (MAF) of Caucasians and Asians is different, stratified analysis was performed based on ethnicity (Caucasian and Asian) in TSA. Regarding sample size estimation, type I error, power, and heterogeneity were set to 0.05%, 0.8%, and 80%, respectively. A review of previous literature showed that the odds ratio (OR) of gene mutations and osteoporosis was approximately 1.5. A 1,000-point genome database was used as a reference for the MAFs of various loci, and an allele model was used for inheritance mode analysis. A random-effects model was used to combine the sample sizes of studies, and I^2^ > 50% was defined as high heterogeneity between the included publications.

### Availability of data and materials

All data generated or analyzed during this study are included in this published article.

## RESULTS

We searched meta-analysis papers, and we found 203 papers from PubMed, Embase, and Cochrane databases. After excluding 164 papers because they were not meta-analysis studies, 39 papers were included in the study along with 21 gene loci. Figure 1 shows the literature search process. Among the 21 gene loci, 17 and 15 were related to Caucasian and Asian populations, respectively. These gene loci were stratified by ethnicity for TSA. The sample size estimation results are shown in Supplementary Figures 1–32.

**Figure 1:**
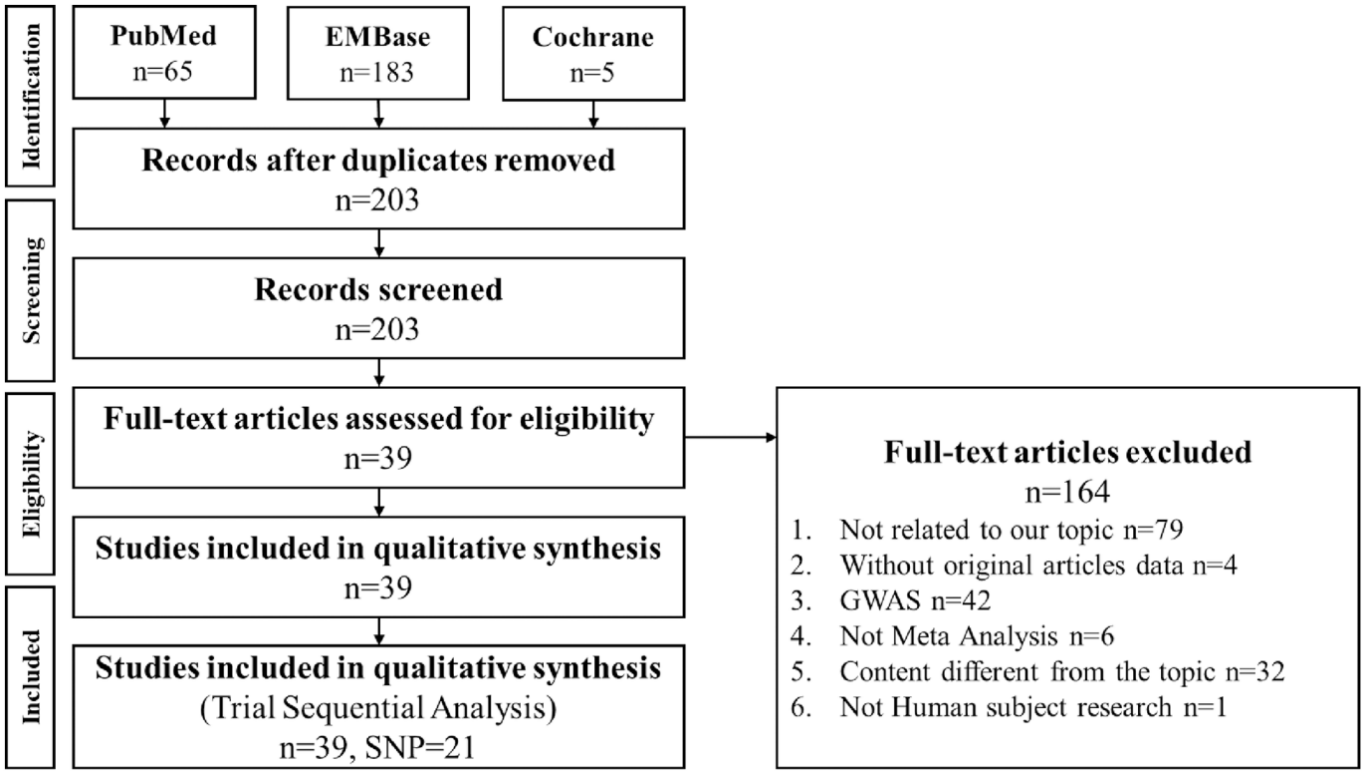
**PRISMA flowchart of decisive gene strategy.** Literature search results of the PubMed, Embase, and Cochrane databases were compiled and analyzed. Among 203 papers, 164 were excluded as nonrelevant to meta-analysis, and 39 papers were included in this meta-analysis, including 21 gene loci.

Table 1 shows that in the Caucasian population, the cumulative sample sizes for eight gene loci, namely, vitamin D receptor (VDR) ApaI (rs7975232), VDR BsmI (rs1544410), interleukin 6 (IL6) G174C (rs1800795), collagen type I alpha 1 (COL1A1) 1245GT (rs1800012), estrogen receptor 1 (ESR1) PvuII (rs2234693), ESR1 XbaI (rs9340799), estrogen receptor 2 (ESR2) RsaI (rs1256049), and osteoprotegerin (OPG) T245G (rs3134069), were sufficient for a conclusion of noncorrelation with osteoporosis. More cases were required for the other nine gene loci, namely, VDR FokI (rs2228570), VDR TaqI (rs731236), transforming growth factor-β1 (TGF β1) T869C (rs1800470), low-density lipoprotein receptor-related proteins 5 (LRP5) (rs3736228), ESR1 G2014A (rs2228480), ESR2 AluI (rs4986938), OPG T950C (rs2073617), OPG A163G (rs3102735), and OPG G1181C (rs2073618), before a definite conclusion could be made on their correlation with osteoporosis.

**Table 1:** Association of candidate gene SNPs with osteoporosis among Caucasian population.

	**Gene** **SNP**	**rs** **number**	**Article** **quantity**	**Major** **minor**	**MAF**	**GTEx** **p- value** **(Blood)**	**Accumulated** **samples**	**TSA result**	**Test of** **association**		**Test of** **heterogeneity**	
									**OR** **(95%CI)**	**p-** **value**	**I^2^**	**p-** **value**
01	VDR ApaI	rs7975232	7	C/A	45%	0.50	1728	Determine the mutation is not significantly associated with osteoporosis.	0.9 (0.72 to 1.36)	0.9579	72%	0.0017
02	VDR BsmI	rs1544410	16	C/T	40%	0.90	3620	Determine the mutation is not significantly associated with osteoporosis.	0.92 (0.76 to 1.11)	0.3841	64%	0.0002
03	IL6 G174C	rs1800795	9	G/C	42%	1.6e-3	7536	Determine the mutation is not significantly associated with osteoporosis.	0.94 (0.87 to 1.01)	0.0696	0%	0.4418
04	COL1A1 1245GT	rs1800012	8	C/A	19%	0.24	1633	Determine the mutation is not significantly associated with osteoporosis.	1.27 (0.71 to 2.27)	0.4195	88%	0.0000
05	ESR1 PvuII	rs2234693	7	T/C	42%	0.26	1726	Determine the mutation is not significantly associated with osteoporosis.	1.06 (0.75 to 1.50)	0.7298	82%	0.0000
06	ESR1 XbaI	rs9340799	7	A/G	31%	0.71	1839	Determine the mutation is not significantly associated with osteoporosis.	0.87 (0.58 to 1.31)	0.5099	88%	0.0000
07	ESR2 RsaI	rs1256049	1	C/T	4%	0.68	380	Determine the mutation is not significantly associated with osteoporosis.	1.30 (0.81 to 2.10)	0.2740	0%	0.0000
08	OPG T245G	rs3134069	2	A/C	4%	NA	596	Determine the mutation is not significantly associated with osteoporosis.	0.79 (0.06 to 10.28)	0.8551	68%	0.0775
09	VDR FokI	rs2228570	2	G/A	38%	0.18	320	Still need to accumulate 2305 samples to determine.	0.96 (0.69 to 1.34)	0.8124	0%	0.9999
10	VDR TaqI	rs731236	5	A/G	40%	1.9e-3	1056	Still need to accumulate 1547 samples to determine.	1.34 (0.94 to 1.92)	0.1100	68%	0.0141
11	TGF_β1 T869C	rs1800470	3	G/A	38%	2.2e-8	972	Still need to accumulate 1656 samples to determine.	1.03 (0.58 to 1.83)	0.9171	70%	0.0339
12	LRP5	rs3736228	2	C/T	13%	0.42	481	Still need to accumulate 4459 samples to determine.	1.5 (1.08 to 2.07)	0.0149	0%	0.6337
13	ESR1 G2014A	rs2228480	1	G/A	17%	0.34	570	Still need to accumulate 3448 samples to determine.	0.63 (0.44 to 0.90)	0.0117	0%	0.0000
14	ESR2 AluI	rs4986938	3	C/T	38%	2.1e-3	1097	Still need to accumulate 1531 samples to determine.	1.23 (0.58 to 2.57)	0.5899	94%	0.0000
15	OPG T950C	rs2073617	1	G/A	49%	NA	555	Still need to accumulate 1395 samples to determine.	0.93 (0.73 to 1.17)	0.5118	0%	0.0000
16	OPG A163G	rs3102735	2	T/C	13%	NA	624	Still need to accumulate 4316 samples to determine.	1.49 (1.11 to 2.00)	0.0079	0%	0.4996
17	OPG G1181C	rs2073618	1	G/C	47%	NA	555	Still need to accumulate 2625 samples to determine.	0.87 (0.70 to 1.10)	0.2523	0%	0.0000

MAF, Minor allele frequency, data from http://grch37.ensembl.org/index.html

GTEx, Genotype- Tissue Expression.

Table 2 shows that in the Asian population, the cumulative sample sizes for five gene loci, namely, VDR FokI (rs2228570), TGF β1 T869C (rs1800470), insulin-like growth factor 1 (IGF1) (rs2288377), IGF1 (rs35767), and ESR2 RsaI (rs1256049), were sufficient for a conclusion of correlation with osteoporosis. Among these loci, VDR FokI (rs2228570, OR = 1.44, 95% confidence interval (95% CI) = 1.22–1.70), TGF β1 T869C (rs1800470, OR = 1.35, 95% CI =1.10–1.65), IGF1 (rs2288377, OR = 1.44, 95% CI = 1.28–1.62), and IGF1 (rs35767, OR = 1.20, 95% CI = 1.06–1.36) were identified as risk factors for osteoporosis, whereas ESR2 RsaI (rs1256049, OR = 0.69, 95% CI = 0.59–0.81) was identified as a protective factor for osteoporosis. The sample sizes for four gene loci, namely, COL1A1 1245GT (rs1800012), IGF1 (rs5742612), IL6 G174C (rs1800795), and ESR1 XbaI (rs9340799), were sufficient for a conclusion of noncorrelation with osteoporosis. More cases were required for the remaining six gene loci, namely, ESR1 PvuII (rs2234693), VDR ApaI (rs7975232), VDR BsmI (rs1544410), COL1A1 1997GT (rs1107946), ESR1 G2014A (rs2228480), and ESR2 AluI (rs4986938), before a definite conclusion could be made on their correlation with osteoporosis.

**Table 2:** Association of candidate gene SNPs with osteoporosis among Asian population.

	**Gene**	**rs** **number**	**Article** **quantity**	**Major** **minor**	**MAF**	**GTEx** **p- value** **(Blood)**	**Accumulated** **samples**	**TSA result**	**Test of** **association**		**Test of** **heterogeneity**	
									**OR** **(95%CI)**	**p-** **value**	**I^2^**	**p-** **value**
01	VDR FokI	rs2228570	3	G/A	42%	0.18	1300	Determine the mutation is significantly associated with osteoporosis. (Risk)	1.44 (1.22 to 1.70)	<0.0001	0%	0.6276
02	TGF_β1T869C	rs1800470	7	G/A	45%	2.2e-8	3472	Determine the mutation is significantly associated with osteoporosis.(Risk)	1.35 (1.10 to 1.65)	0.0047	74%	0.0007
03	IGF1	rs2288377	6	A/T	29%	0.94	4029	Determine the mutation is significantly associated with osteoporosis.(Risk)	1.44 (1.28 to 1.62)	<0.0001	0%	0.5534
04	IGF1	rs35767	7	G/A	37%	0.30	4575	Determine the mutation is significantly associated with osteoporosis.(Risk)	1.20 (1.06 to 1.36)	0.0032	47%	0.0791
05	ESR2 RsaI	rs1256049	1	C/T	40%	0.68	1190	Determine the mutation is significantly associated with osteoporosis. (Protect)	0.69 (0.59 to 0.81)	<0.0001	0%	0.0000
06	COL1A1 1245GT	rs1800012	1	C/A	0.1%	0.24	349	Determine the mutation is not significantly associated with osteoporosis.	0.83 (0.56 to 1.23)	0.3536	0%	0.0000
07	IGF1	rs5742612	6	A/G	29%	0.45	4031	Determine the mutation is not significantly associated with osteoporosis.	1.1 (0.97 to 1.26)	0.1394	0%	0.9564
08	IL6 G174C	rs1800795	1	G/C	0.1%	1.6e-3	318	Determine the mutation is not significantly associated with osteoporosis.	0.61 (0.05 to 7.28)	0.6921	0%	0.0000
09	ESR1 XbaI	rs9340799	7	A/G	19%	0.71	3123	Determine the mutation is not significantly associated with osteoporosis.	0.86 (0.39 to 1.89)	0.7093	97%	0.0000
10	ESR1 PvuII	rs2234693	7	T/C	40%	0.26	3010	Still need to accumulate 2075 samples to determine.	0.82 (0.55 to 1.22)	0.3210	90%	0.0000
11	VDR ApaI	rs7975232	7	C/A	29%	0.50	1804	Still need to accumulate 1284 samples to determine.	1.21 (0.81 to 1.80)	0.3442	81%	0.0000
12	VDR BsmI	rs1544410	19	C/T	6%	0.90	2473	Still need to accumulate 7345 samples to determine.	1.01 (0.64 to 1.60)	0.9525	85%	0.0000
13	COL1A1 1997GT	rs1107946	2	C/A	30%	0.08	580	Still need to accumulate 2290 samples to determine.	1.21 (0.87 to 1.67)	0.2564	25%	0.2498
14	ESR1 G2014A	rs2228480	3	G/A	20%	0.34	798	Still need to accumulate 2764 samples to determine.	1.00 (0.23 to 4.46)	0.9955	97%	0.0000
15	ESR2 AluI	rs4986938	1	C/T	13%	2.1e-3	1303	Still need to accumulate 4805 samples to determine.	1.31 (1.05 to 1.64)	0.0164	0%	0.0000

MAF, Minor allele frequency, data from http://grch37.ensembl.org/index.html

GTEx, Genotype-Tissue Expression.

Table 3 shows that in the Caucasian population, there was high heterogeneity between the collected papers for nine gene loci, namely, VDR ApaI (rs7975232), VDR BsmI (rs1544410), COL1A1 1245GT (rs1800012), ESR1 PvuII (rs2234693), ESR1 XbaI (rs9340799), OPG T245G (rs3134069), VDR TaqI (rs731236), TGF β1 T869C (rs1800470), and ESR2 AluI (rs4986938). Meanwhile, there was low heterogeneity between the collected papers for eight gene loci, namely, IL6 G174C (rs1800795), ESR2 RsaI (rs1256049), VDR FokI (rs2228570), LRP5 (rs3736228), ESR1 G2014A (rs2228480), OPG T950C (rs2073617), OPG A163G (rs3102735), and OPG G1181C (rs2073618). Table 4 shows that in the Asian population, there was high heterogeneity between the collected papers for six gene loci, namely, TGF β1 T869C (rs1800470), ESR1 XbaI (rs9340799), ESR1 PvuII (rs2234693), VDR ApaI (rs7975232), VDR BsmI (rs1544410), and ESR1 G2014A (rs2228480). Besides, there was low heterogeneity between the collected papers for nine gene loci, namely, VDR FokI (rs2228570), IGF1 (rs2288377), IGF1 (rs35767), ESR2 RsaI (rs1256049), COL1A1 1245GT (rs1800012), IGF1 (rs5742612), IL6 G174C (rs1800795), COL1A1 1997Gt (rs1107946), and ESR2 AluI (rs4986938).

**Table 3:** Cross table between (risk of osteoporosis with gene SNPs) and (literatures heterogeneity) among Caucasian population.

		**Association**			
		**Risk**	**Protect**	**Not association**	**Still need to accumulate samples**
Heterogeneity	High			rs7975232 rs1544410 rs1800012 rs2234693 rs9340799 rs3134069	rs731236 rs1800470 rs4986938
	Low			rs1800795 rs1256049	rs2228570 rs3736228 rs2228480 rs2073617 rs3102735 rs2073618

**Table 4:** Cross table between (risk of osteoporosis with gene SNPs) and (literatures heterogeneity) among Asian population.

		**Association**			
		**Risk**	**Protect**	**Not association**	**Still need to accumulate samples**
Heterogeneity	High	rs1800470		rs9340799	rs2234693 rs7975232 rs1544410 rs2228480
	Low	rs2228570 rs2288377 rs35767	rs1256049	rs1800012 rs5742612 rs1800795	rs1107946 rs4986938

## DISCUSSION

Presently, GWAS is a paramount research technique for understanding the correlation between genetic factors and a disease. This technique can scan millions of SNPs at once; however, the lack of a hypothesis analysis process prevents the discussion of possibly related SNPs from a biological pathway perspective, thereby causing the missing heritability problem. Although the conventional method of genetic association can be used to identify disease candidate genes through biological pathways and to overcome the deficiencies of GWAS, it usually has an inadequately small sample size and examines only a few SNPs. Therefore, the conventional method does not provide a comprehensive understanding of the correlation between a specific gene fragment and disease. Recently, performing meta-analyses on these genetic correlation studies to improve the inherent deficiencies has become a well-known method for increasing the evidence level. These studies solved the lack of resource problem by accumulating samples. However, only a single gene or locus can be analyzed, and the complete correlation between genetic factors and disease cannot be provided. Additionally, continuous meta-analysis can increase the type I error’s probability. Besides, the original conclusions may change when new studies on the same subject are reported and are retested [7–9]. Therefore, a statistical method is required to estimate the final cumulative sample size required when meta-analysis studies are conducted and to determine when to stop adding new studies for meta-analysis. TSA can be used to solve this problem by stopping the samples’ continuous accumulation in the conventional meta-analysis on time and by using images to decide whether to stop the sample accumulation [10].

To avoid problems that may be encountered in GWAS, genetic association studies, and meta-analyses, we developed a DGS to screen for disease-related gene loci. In this strategy, meta-analysis papers were first searched to avoid the previously discussed problems, such as the failure to explore related SNPs from a biological pathway perspective in GWAS, small sample sizes of genetic association studies, and single gene or locus analysis in meta-analyses. TSA was employed to statistically analyze the cumulative sample sizes of all gene loci found, and image results were employed to determine whether a definite conclusion can be drawn for gene loci to show its disease association, and thus to stop further sample accumulation. After applying DGS, we found that five gene locus polymorphisms in the Asian population were associated with osteoporosis: VDR FokI (rs2228570), IGF1 (rs2288377), IGF1 (rs35767), TGF β1 T869C (rs1800470), and ESR2 RsaI (rs1256049).

VDR is a major receptor that regulates vitamin D absorption in humans and is associated with osteocyte function and osteoclast differentiation [11, 12]. Gene polymorphisms in VDR affect the expression and transcription of genes associated with osteogenesis and calcium absorption (such as osteocalcin and calcium-binding proteins) [13]. Importantly, such gene polymorphisms affect VDR expression and function, thereby influencing the risk of developing osteoporosis [14]. The VDR FokI variant is located in exon 2 of the VDR gene; this causes the loss of the ATG translation initiation region, resulting in a shorter and more active VDR protein, which plays a crucial role in message stability and posttranscriptional processes [15, 16]. In 2006, Zintzaras et al. [17] performed a meta-analysis on the correlation between VDR gene polymorphisms and osteoporosis. They found that VDR FokI gene polymorphism did not significantly correlate with osteoporosis (OR = 1.17, 95% CI = 0.76–1.80) and that other loci on the VDR gene did not significantly correlate with osteoporosis. In 2013, a meta-analysis on menopausal women by Wang et al. [18] showed that VDR FokI gene polymorphism significantly correlated with reduced bone mineral density (BMD) (standard mean deviation (SMD) = 0.68, 95% CI = 0.34–1.03), but other loci on the VDR gene were not significantly correlated. However, past meta-analyses were not stratified by ethnicity. In this study, when the DGS was used for stratification by ethnicity, it became evident that VDR FokI was not correlated with osteoporosis in Caucasians, and TSA results showed that more cases were required to obtain a definite conclusion; conversely, the DGS showed that VDR FokI significantly correlated with osteoporosis in Asians. Additionally, the TSA results confirmed that a definite conclusion on this correlation could be made.

IGF1 affects osteocytes’ growth, division, and apoptosis and is considered a critical factor affecting the expression of growth hormones during bone growth and mineralization [19, 20]. IGF1 also promotes osteoblasts’ growth and apoptosis *in vivo* via the phosphoinositide 3-kinase (PI3K) pathway [21]*.* Additionally, IGF1 can induce strong proliferation and osteogenic differentiation in bone marrow mesenchymal stem cells via Wnt/β-catenin and Akt signaling pathways [22, 23]. In 2017, Chen et al. [24] performed a meta-analysis on the correlation between IGF1 and osteoporosis in a Chinese population and found that rs35767 in IGF1 was associated with risks of osteoporosis (OR = 1.31, 95% CI = 1.18–1.47, P value < 0.001), whereas other loci (e.g., rs2288377 and rs5742612) in IGF1 were not significantly correlated with osteoporosis. In 2018, Gao et al. [14] performed a meta-analysis on menopausal Han Chinese women and obtained similar results on the correlation between IGF1 and osteoporosis. Regarding this study of rs35767 loci in IGF1, TSA results on Asians were similar to other previous meta-analysis results, except for rs2288377, which differed from previous studies. The reason could be the lower sample sizes of previous meta-analyses, and this study used the DGS followed by TSA to accumulate previous samples. In addition to having a larger sample size, a definite conclusion could be drawn regarding the significant correlation between rs2288377 and osteoporosis.

TGF β1 is a potent cytokine and bone-derived factor [25]. In addition to playing a crucial role in osteoblast differentiation, assisting tissue regeneration, and bone remodeling, TGF β1 is associated with osteoclast growth and enhances TNFα-induced osteoclast formation and bone destruction, thereby affecting bone resorption and recovery [26, 27]. In 2015, Sun et al. [28] performed a meta-analysis on postmenopausal women and found that TGF β1 T869C correlated significantly with osteoporosis (OR = 1.18, 95% CI = 1.02–1.36, P value = 0.030). An identical conclusion was drawn when a single ethnicity, Asians, was analyzed (OR = 1.18, 95% CI = 1.01–1.38, P value = 0.043). They also examined TGF β1 T29C, another locus in TGF β1, and the results showed that this locus significantly correlated with osteoporosis. In 2016, Cong et al. [25] performed a meta-analysis to examine the correlation between TGF β1 T869C and osteoporosis. Their results showed that TGF β1 T869C significantly correlated with osteoporosis (OR = 1.26, 95% CI = 1.13–1.41, P value < 0.001). When a single ethnicity, Asians, was analyzed, the results also showed that this locus correlated significantly with osteoporosis (OR = 1.33, 95% CI = 1.18–1.49, P value < 0.001). In this study, TSA results in the DGS showed that TGF β1 T869C correlated significantly with osteoporosis in Asians, which agreed with previous meta-analysis results.

Estrogen causes postmenopausal osteoporosis. After menopause, the reduced ovarian synthesis of estrogen in women results in bone loss, thereby causing osteoporosis [29]. Additionally, estrogen is a regulator of bone metabolism, and a reduction in estrogen concentration results in BMD loss, increased mechanical loading, induced bone remodeling, and postmenopausal osteoporosis development [30, 31]. It has been demonstrated in studies on mice that the functional ESR and Wnt/β-catenin signaling pathways interact in regulating bone mass adaptation in response to mechanical loading [32]. In 2018, Zhu et al. [31] performed a meta-analysis on the correlation between ESR1 and ESR2 gene loci and osteoporosis using menopausal women as study subjects. The study results showed that ESR2 RsaI was not significantly correlated with osteoporosis; however, when stratified analysis by ethnicity was performed, a significant correlation was observed between the loci and osteoporosis in Asians (OR = 0.69, 95% CI = 0.58–0.82, P value < 0.001) but not in Caucasians. In this study, TSA results in the DGS showed that ESR2 RsaI correlated significantly with osteoporosis in Asians, which agreed with previous meta-analysis results.

Although ethnicity was used for stratification before analysis to avoid the possibility of high heterogeneity in this study, results showed that there is still high heterogeneity in many SNPs. The reason may be the presence of gene–environment or gene–gene interactions, which is a problem faced equally in the conventional meta-analysis and DGS used here. For instance, the coat color in pigs is simultaneously affected by KIT and MC1R genes. However, the KIT gene is dominant. When the KIT SNP in pigs is a dominant genotype, coat color is unaffected by the MC1R gene and will be white. Nevertheless, if the KIT SNP is a recessive genotype, coat color will be affected by the MC1R gene [33], which is a classic example of gene–gene interactions. For gene–environment interactions, phenylketonuria only occurs when phenylalanine hydroxylase mutations are present and phenylalanine-containing foods are consumed simultaneously. A single exposure source will not cause phenylketonuria [34]. If interactions are overlooked, many disease-causing genes will be missed, thereby causing missing heritability. Liu et al. [35] highlighted that approximately 80% of missing heritability in Crohn’s disease is due to gene–gene interactions. The reason for high heterogeneity in these SNPs may be the presence of gene–environment or gene–gene interactions.

The employed DGS has the following advantages: First, our method was used to search for candidate osteoporosis-related genes, and we found that five gene locus mutations in Asians correlated with osteoporosis. However, the latest GWAS results on osteoporosis failed to show that these five loci are associated with osteoporosis [36]. Thus, it is evident that our method, in addition to GWAS, can be used with respect to more diseases to search for more disease-related candidate genes and to overcome the missing heritability problem. Second, the DGS used TSA for the statistical analysis of the cumulative sample sizes for the identified gene loci, and the image results were used to verify whether there were enough samples for a definite conclusion or whether a gene locus was associated with the disease, and thus, the sample accumulation could stop. Based on these findings, we recommend that further examination of potential gene–gene and gene–environment interactions should be performed for the nine and six gene loci that have high heterogeneity in Caucasians and Asians, respectively.

The DGS still has some limitations. First, it was applied only on meta-analysis papers during the initial literature search, and this search method overlooked gene loci that were not included in previous meta-analysis studies. Second, only English papers were included when DGS was used to review meta-analysis papers. The impact of these two limitations may be reduced by performing new meta-analyses, searching representative databases relevant to topics of interest, manually searching papers, and analyzing publication bias [37, 38]. Additionally, the DGS can only analyze a single gene or loci and cannot provide a complete correlation between genetic factors and osteoporosis. However, even with the abovementioned limitations, using meta-analysis literature search and TSA, the DGS can still find candidate disease-related genes impossible to be identified via GWAS, overcome the issue of small sample sizes in conventional genetic association studies, and improve on the inability to estimate the samples’ number to be accumulated in meta-analysis.

## CONCLUSIONS

A novel developed DGS can be used to identify gene loci that may be associated with osteoporosis. In this study, we employed this strategy to find five gene loci associated with osteoporosis in Asians. This study’s most important scientific significance is to propose a novel methodology, the DGS, for generating extensive conclusions of current evidence on SNPs and a specific disease. This study demonstrated the DGS application in osteoporosis-related SNP screening. In the future, we will combine experimental or cohort verification to prove that the DGS results are credible and DGS can be applied to other diseases, perhaps to overcome the missing heritability problem, applying disease-related genes in clinical practice, and to provide appropriate disease prevention policies.

## Supplementary Material

Supplementary Figures

Supplementary Table 1
