# Supplementary material for: Decisive gene strategy on osteoporosis: a comprehensive whole-literature-based approach for conclusive candidate gene targets
Source: Aging (Albany NY). 2022 Apr 22;14(8):3484–528. doi: 10.18632/aging.204026 (PMC9085221; doi:10.18632/aging.204026)
Supplement: Supplementary Figures [file aging-14-204026-s001.pdf]

SUPPLEMENTARY FIGURES

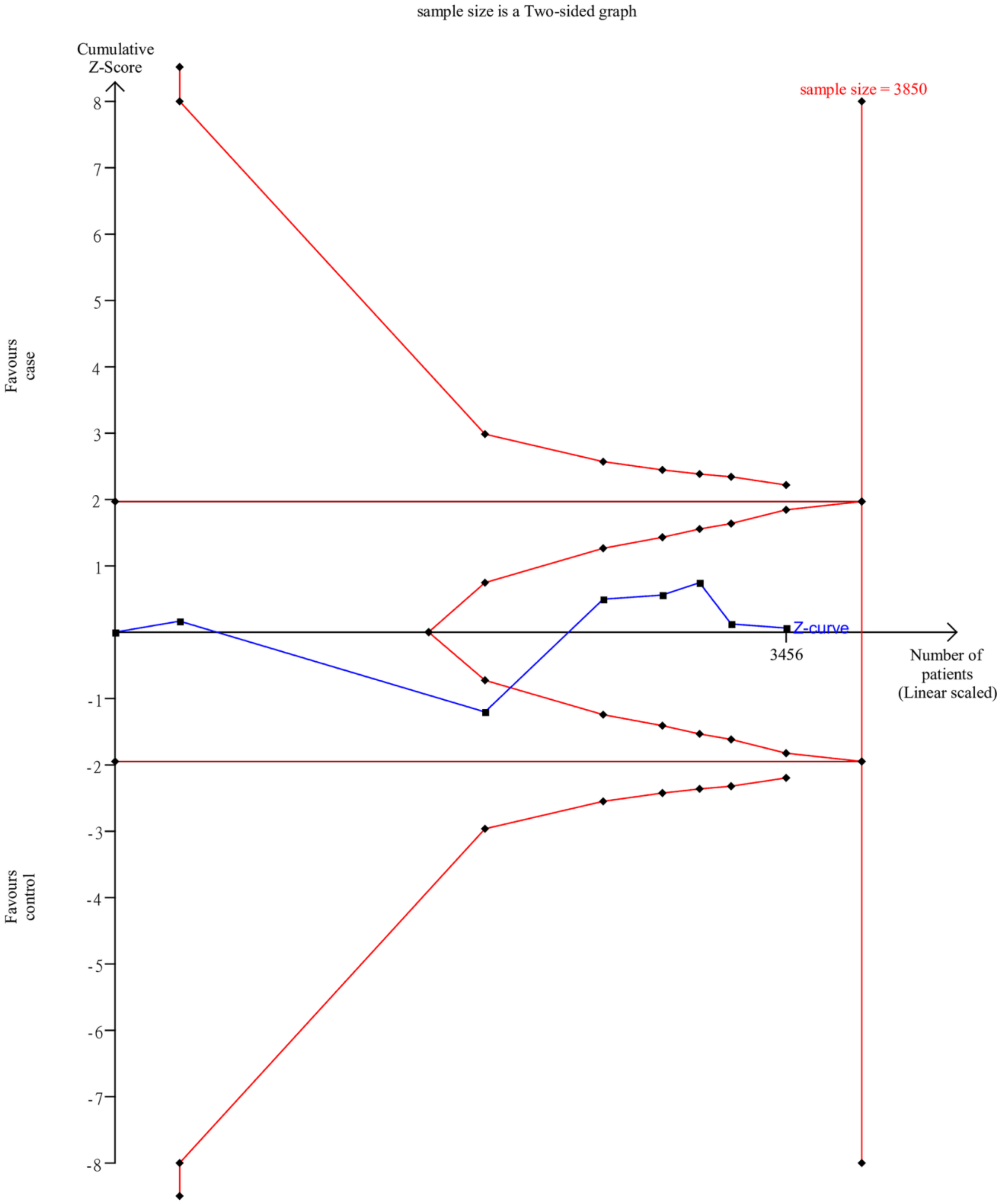

Supplementary Figure 1. Trial sequential analysis (TSA) of the association between rs7975232 polymorphism and the risk of osteoporosis in Caucasians.

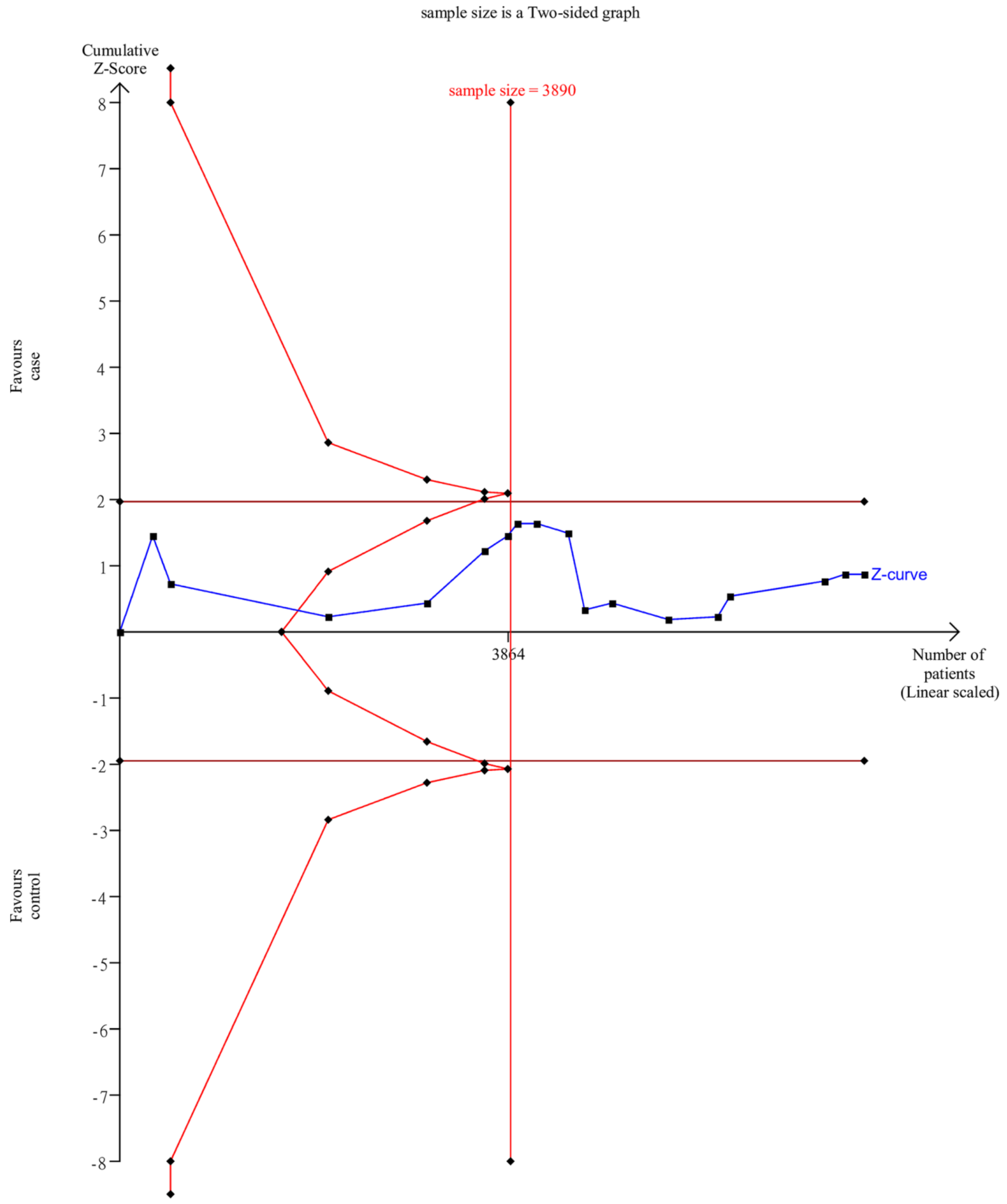

Supplementary Figure 2. TSA of the association between rs1544410 polymorphism and the risk of osteoporosis in Caucasians.

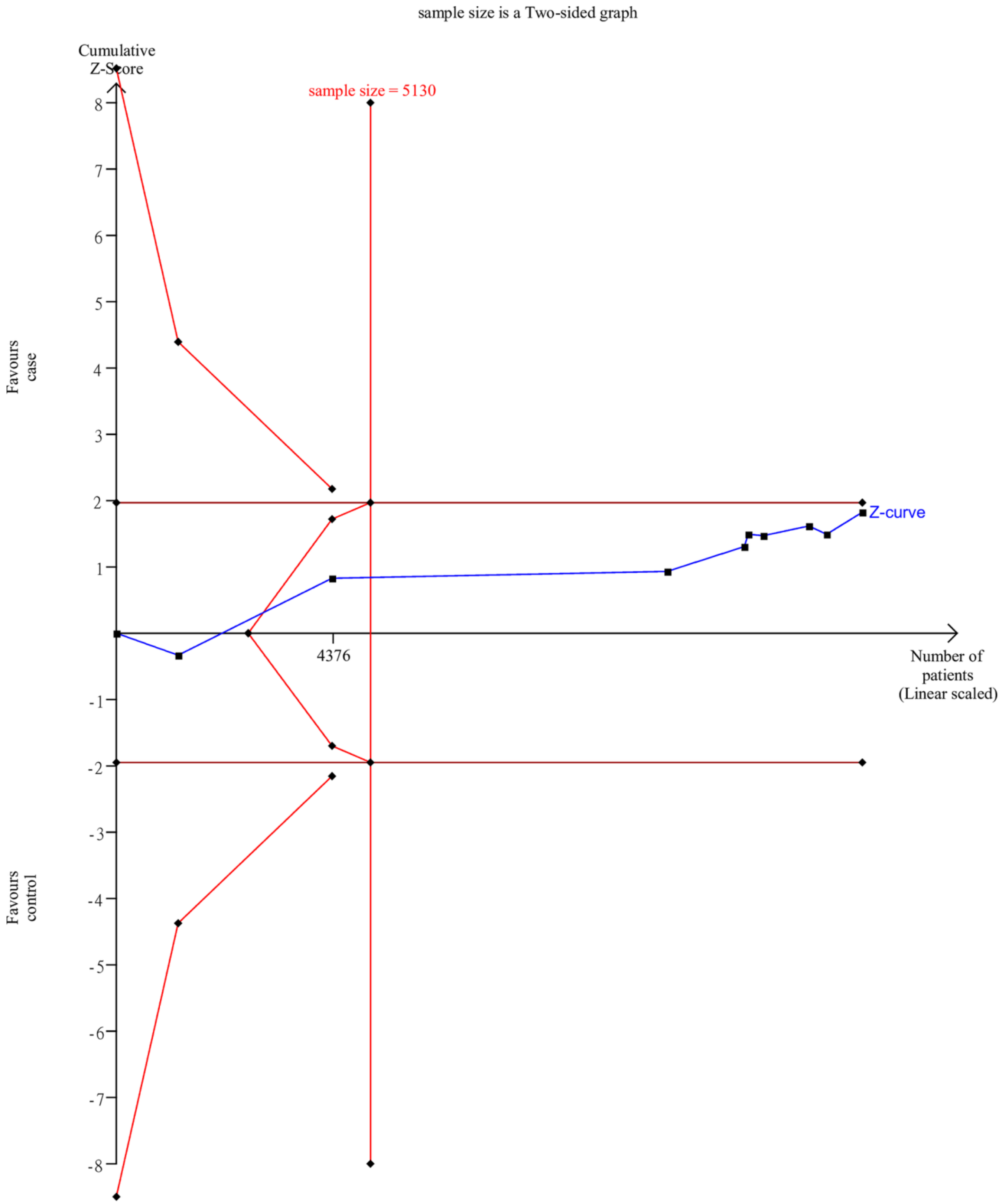

Supplementary Figure 3. TSA of the association between rs1800795 polymorphism and the risk of osteoporosis in Caucasians.

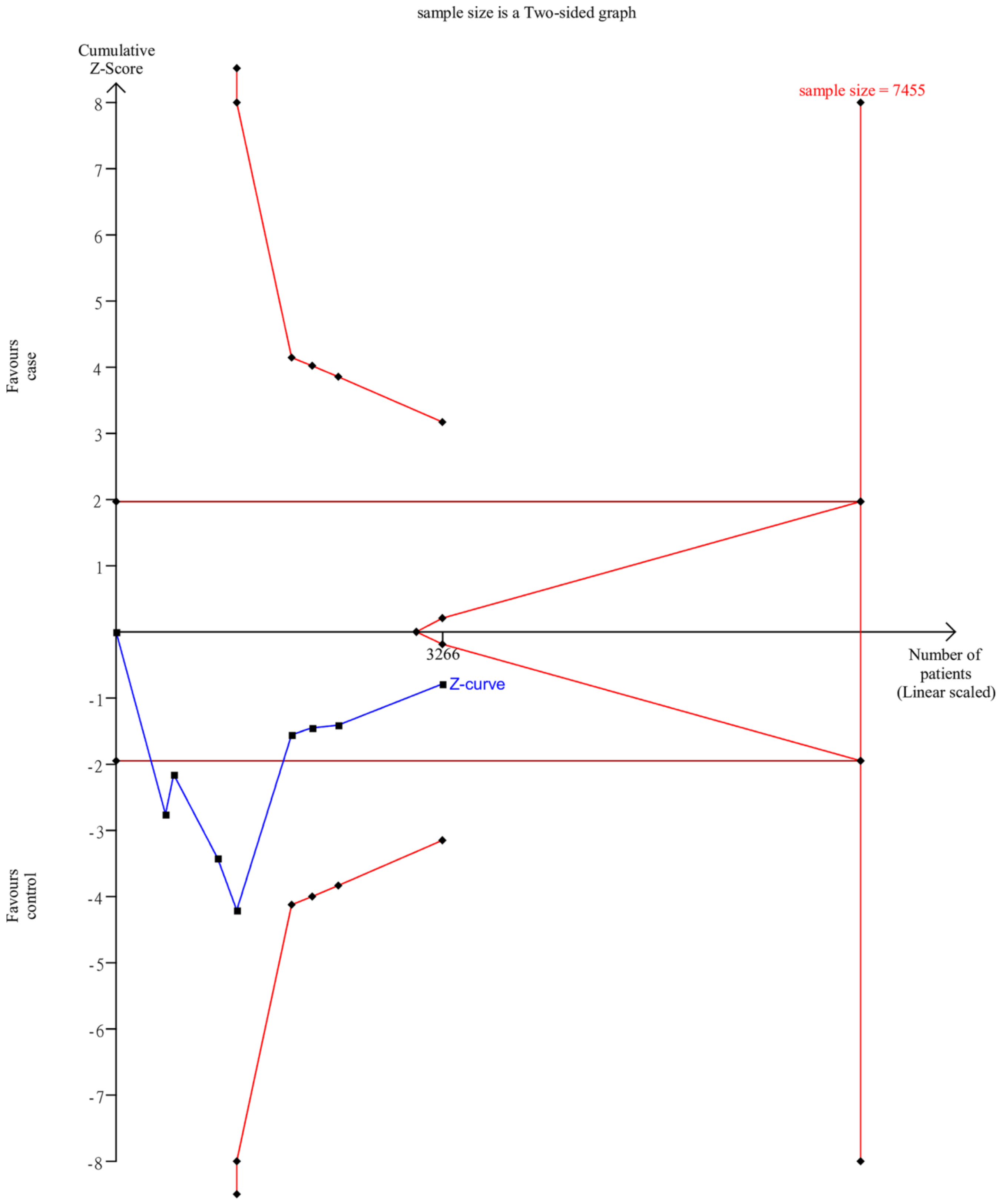

Supplementary Figure 4. TSA of the association between rs1800012 polymorphism and the risk of osteoporosis in Caucasians.

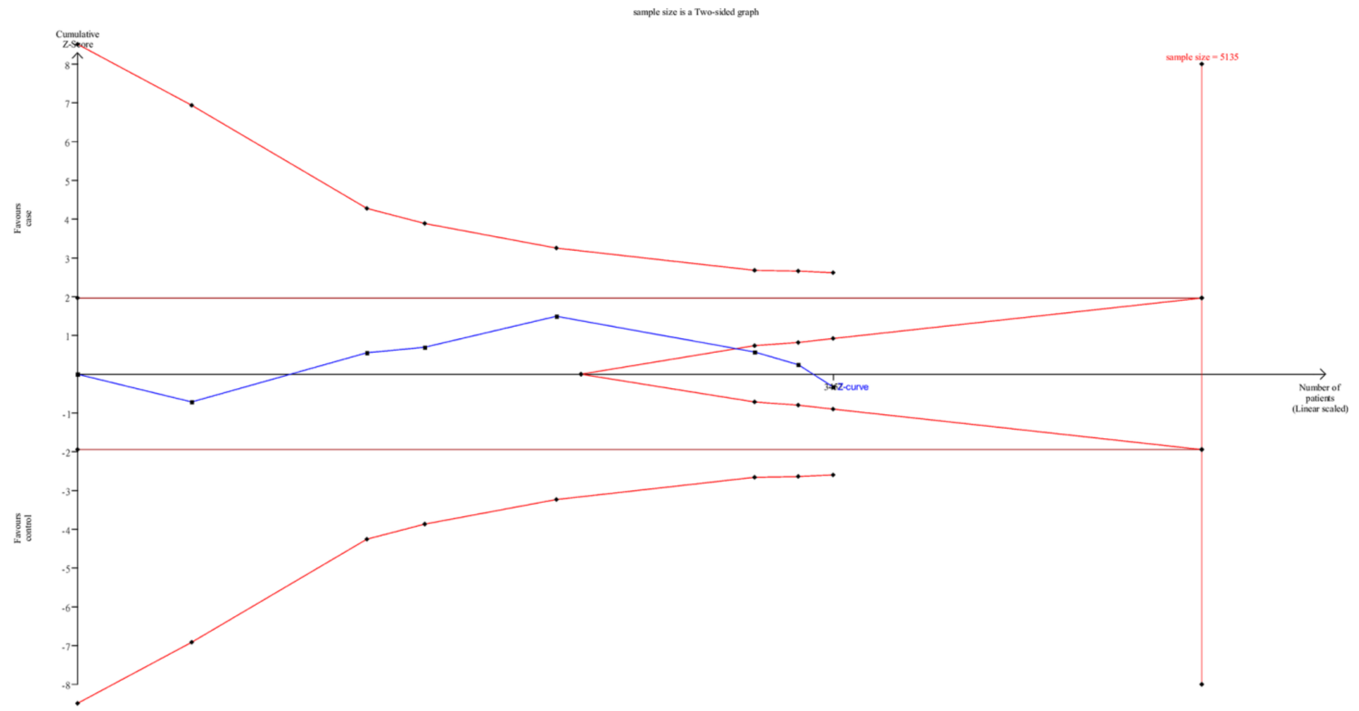

Supplementary Figure 5. TSA of the association between rs2234693 polymorphism and the risk of osteoporosis in Caucasians.

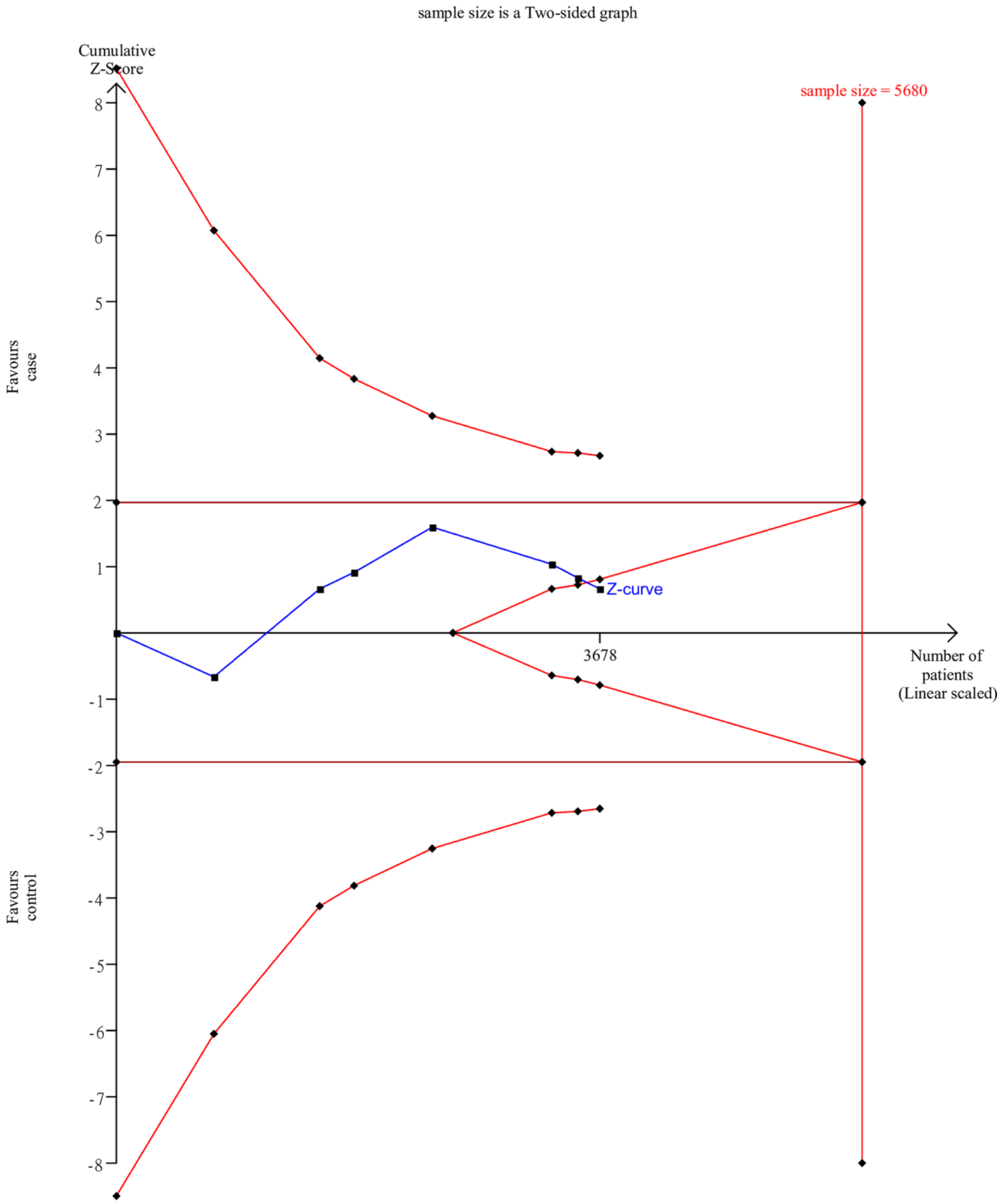

Supplementary Figure 6. TSA of the association between rs9340799 polymorphism and the risk of osteoporosis in Caucasians.

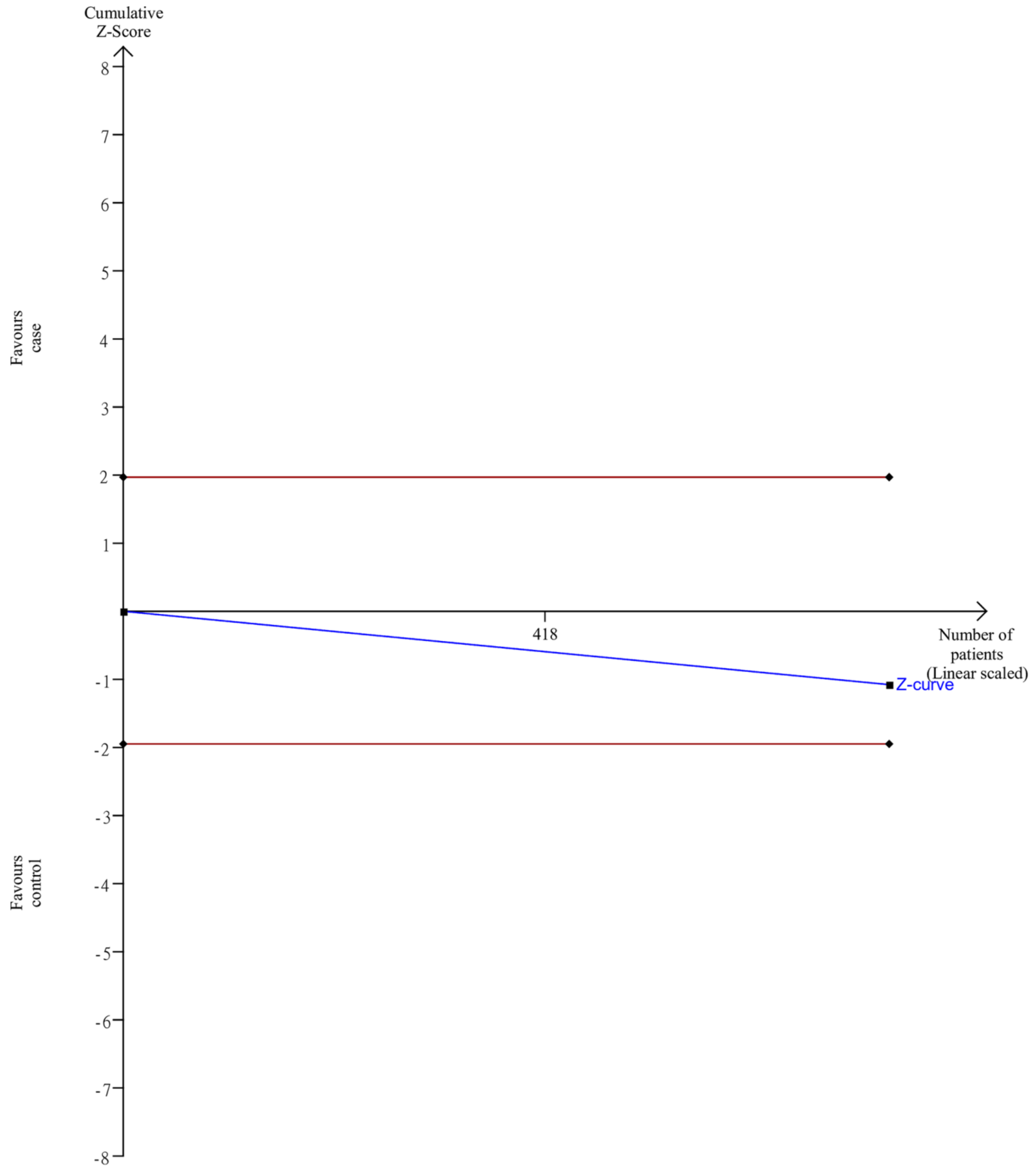

Supplementary Figure 7. TSA of the association between rs1256049 polymorphism and the risk of osteoporosis in Caucasians.

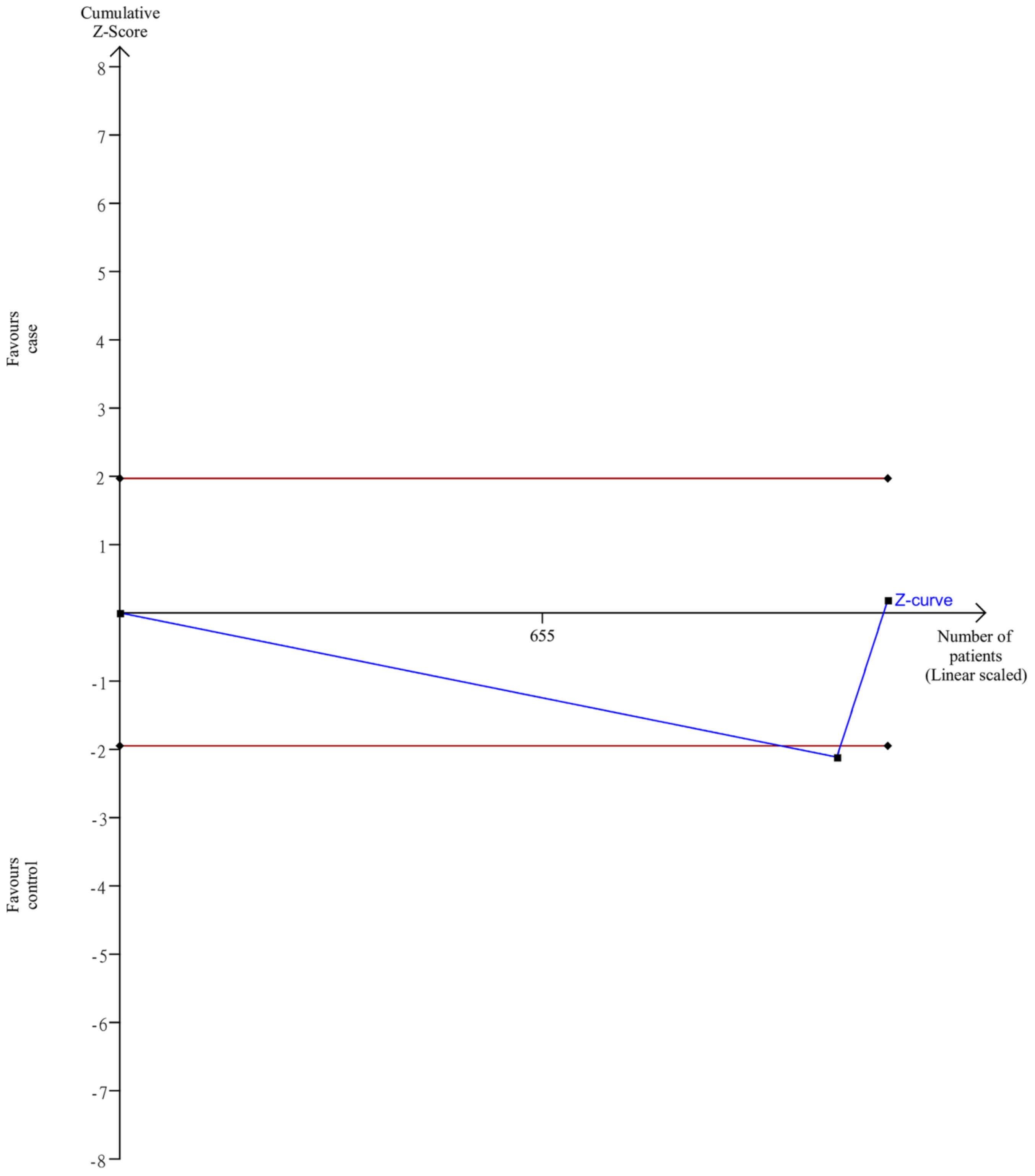

Supplementary Figure 8. TSA of the association between rs3134069 polymorphism and the risk of osteoporosis in Caucasians.

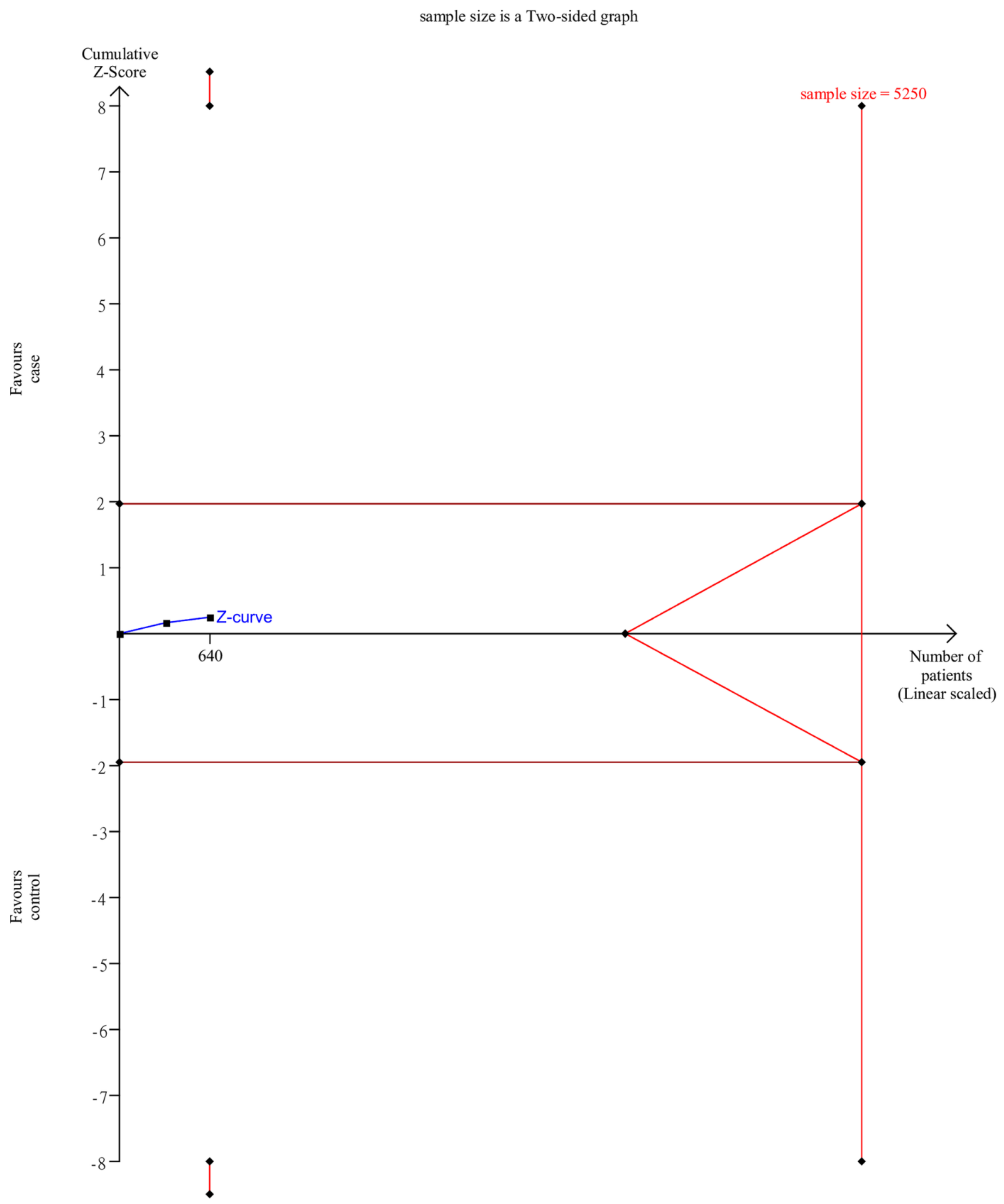

Supplementary Figure 9. TSA of the association between rs2228570 polymorphism and the risk of osteoporosis in Caucasians.

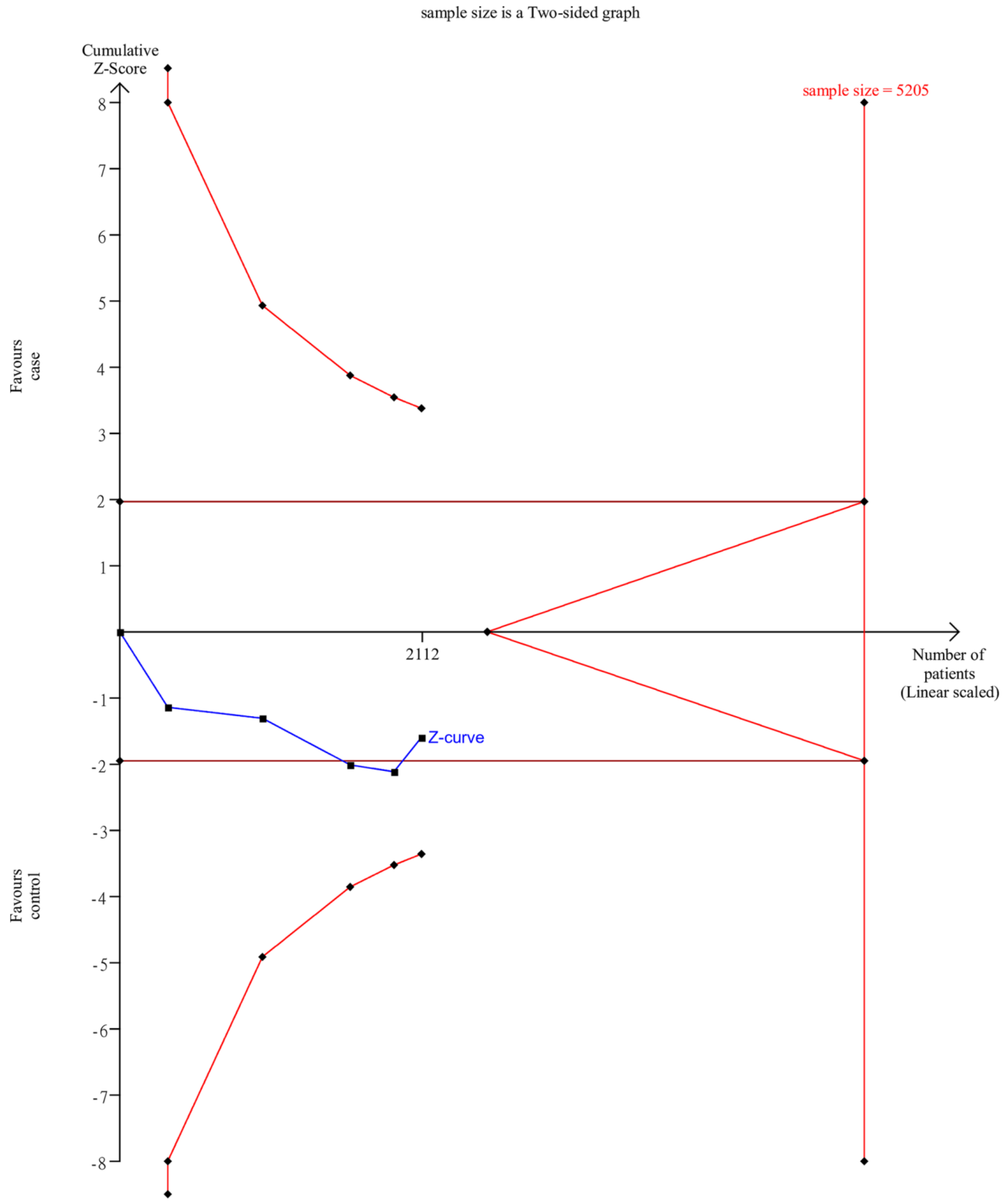

Supplementary Figure 10. TSA of the association between rs731236 polymorphism and the risk of osteoporosis in Caucasians.

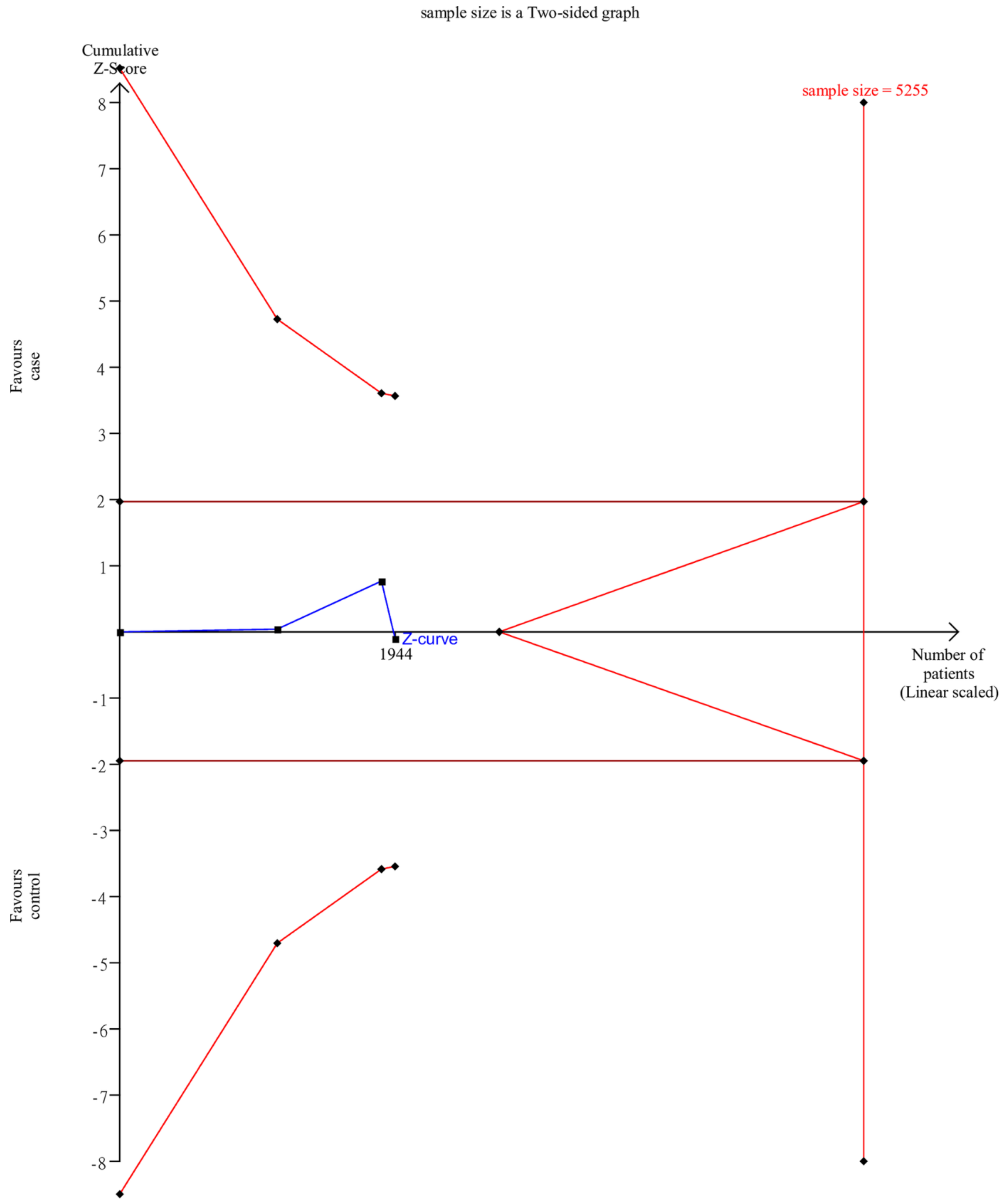

Supplementary Figure 11. TSA of the association between rs1800470 polymorphism and the risk of osteoporosis in Caucasians.

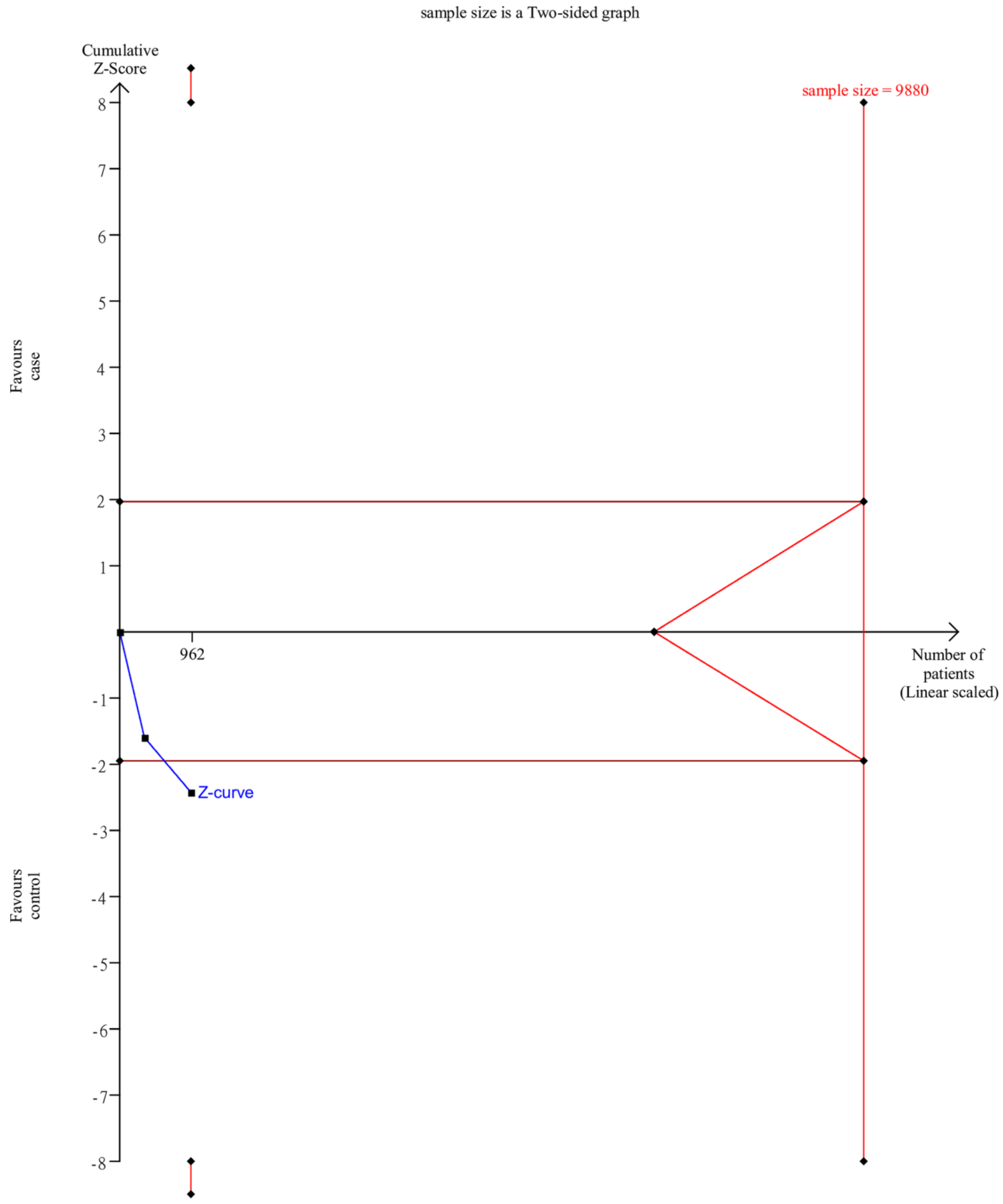

Supplementary Figure 12. TSA of the association between rs3736228 polymorphism and the risk of osteoporosis in Caucasians.

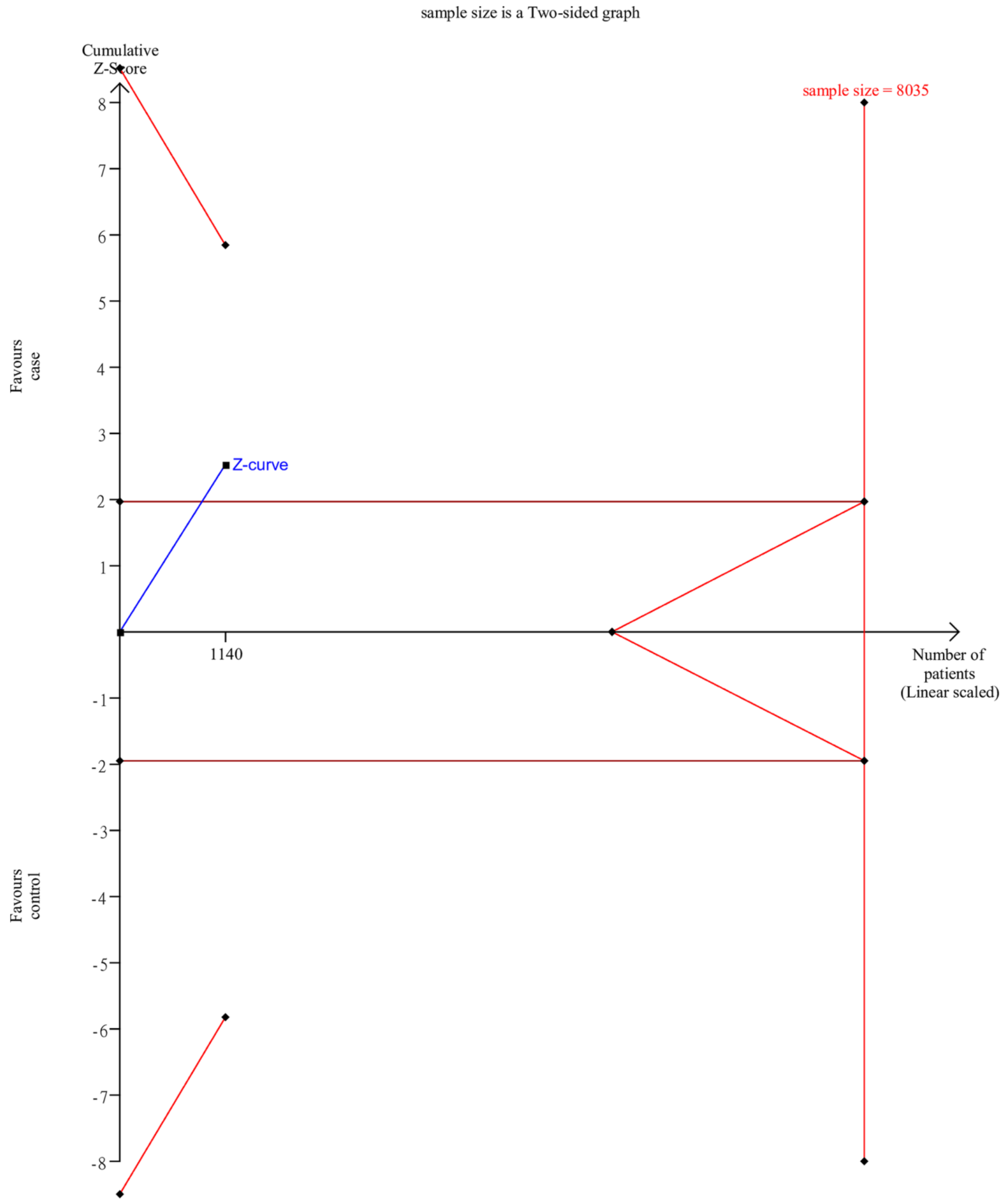

Supplementary Figure 13. TSA of the association between rs2228480 polymorphism and the risk of osteoporosis in Caucasians.

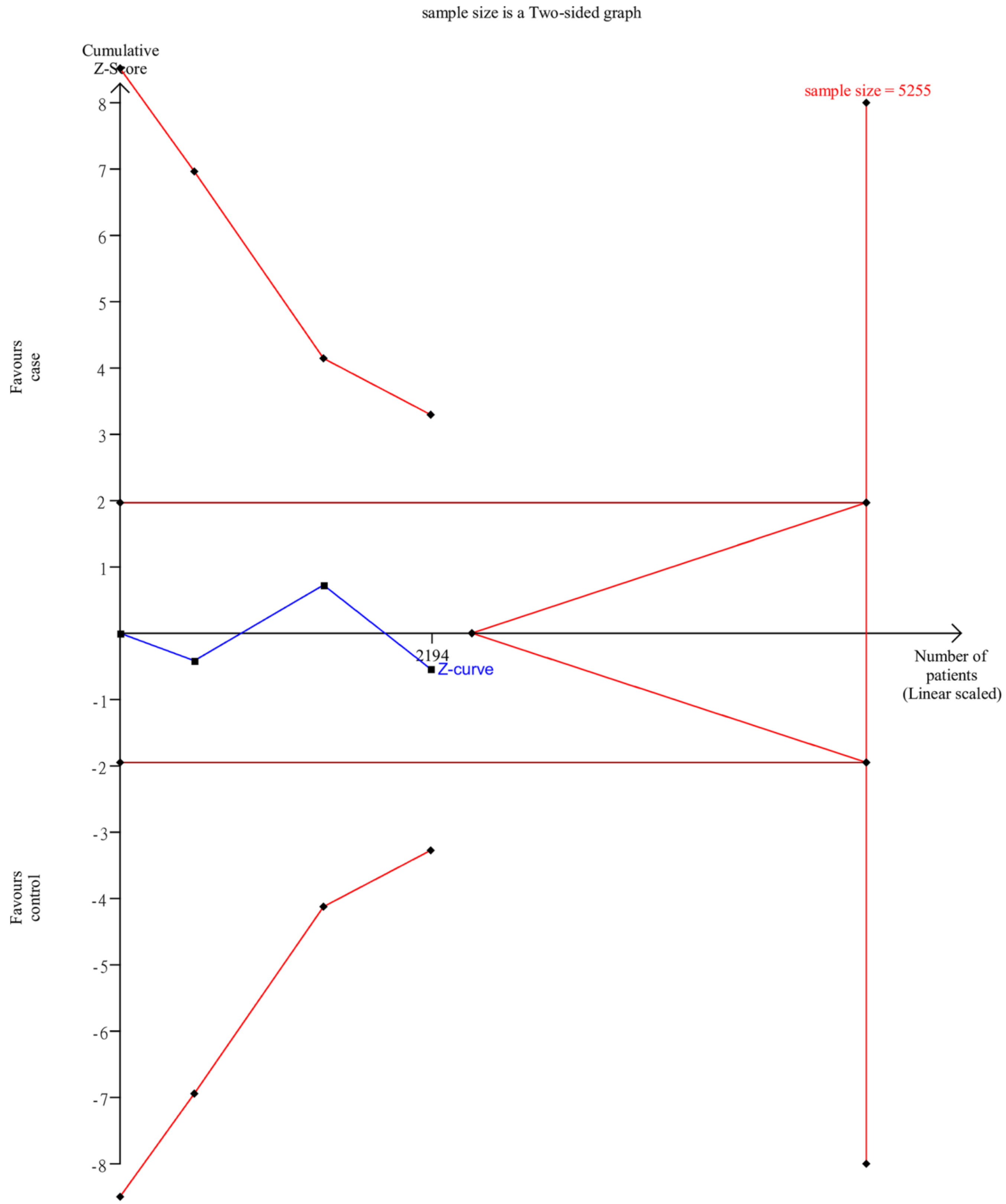

Supplementary Figure 14. TSA of the association between rs4986938 polymorphism and the risk of osteoporosis in Caucasians.

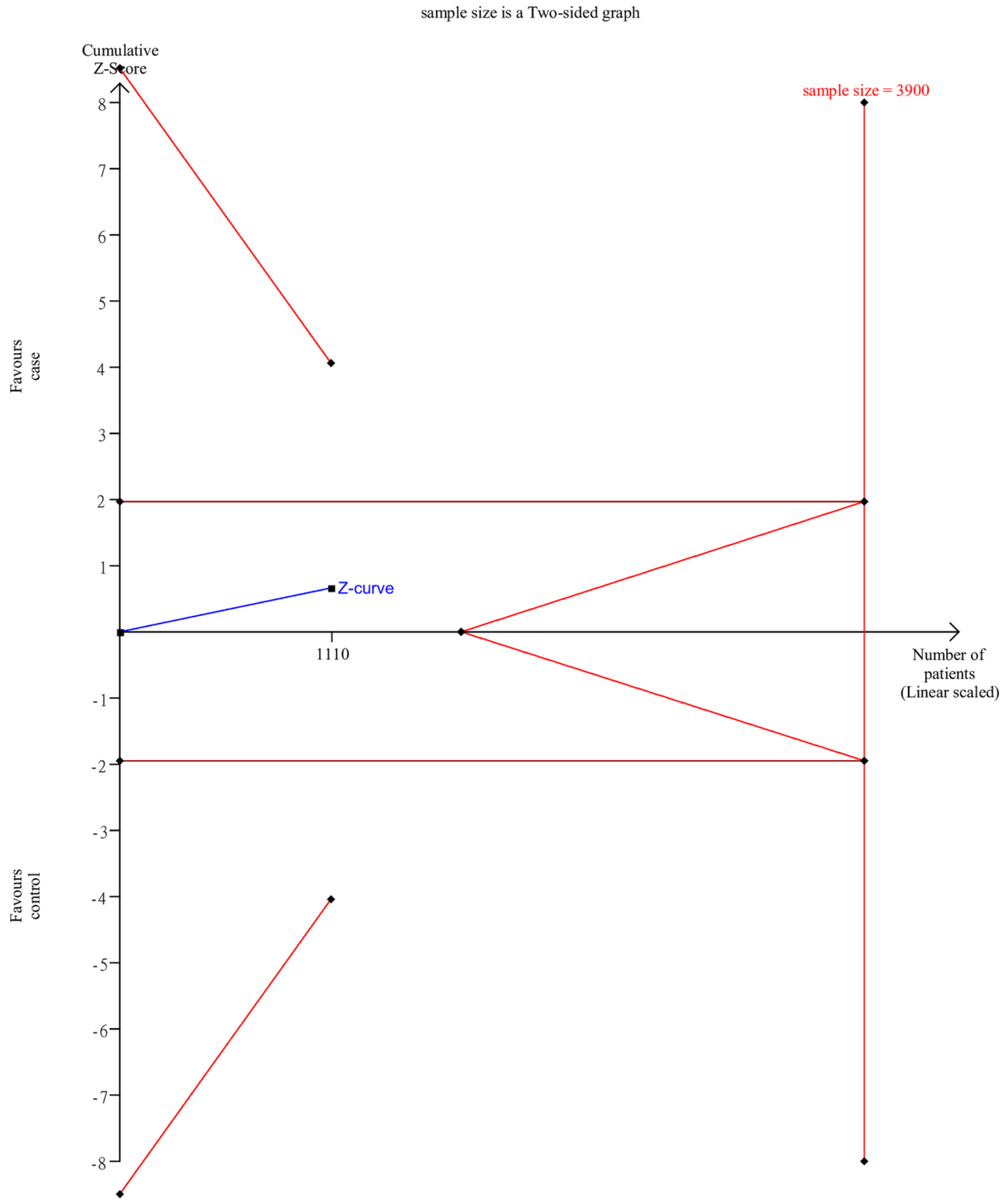

Supplementary Figure 15. TSA of the association between rs2073617 polymorphism and the risk of osteoporosis in Caucasians.

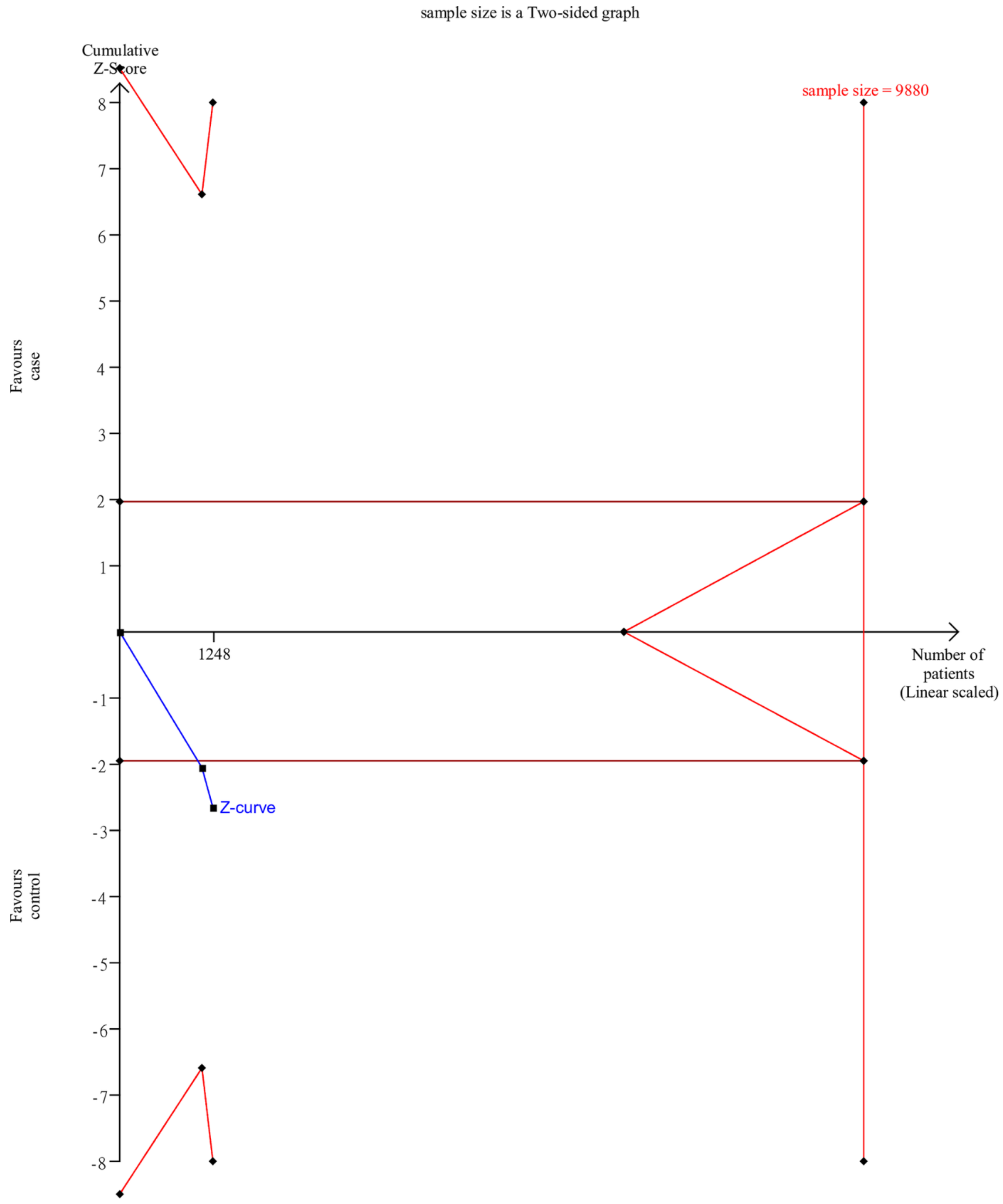

Supplementary Figure 16. TSA of the association between rs3102735 polymorphism and the risk of osteoporosis in Caucasians.

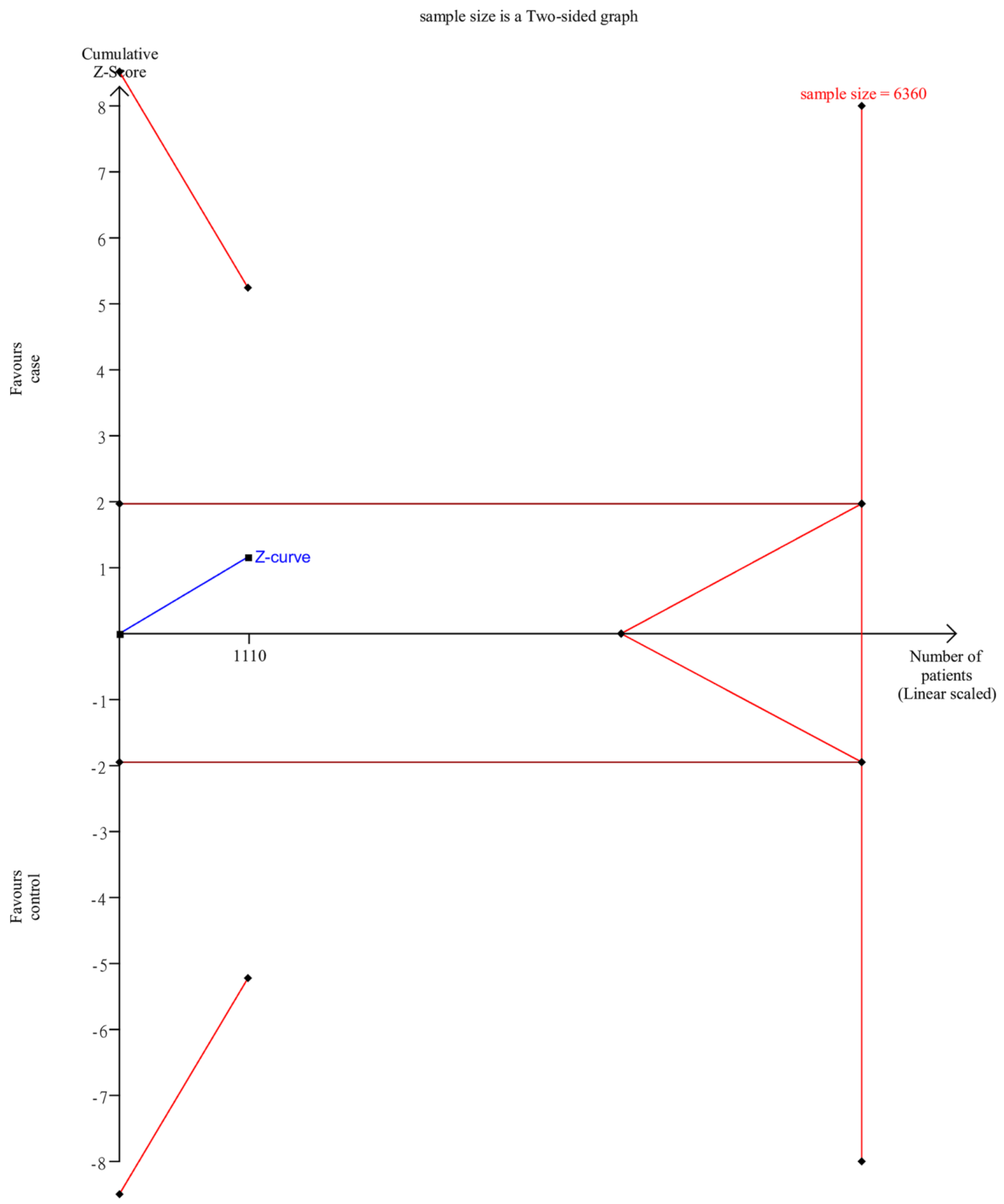

Supplementary Figure 17. TSA of the association between rs2073618 polymorphism and the risk of osteoporosis in Caucasians.

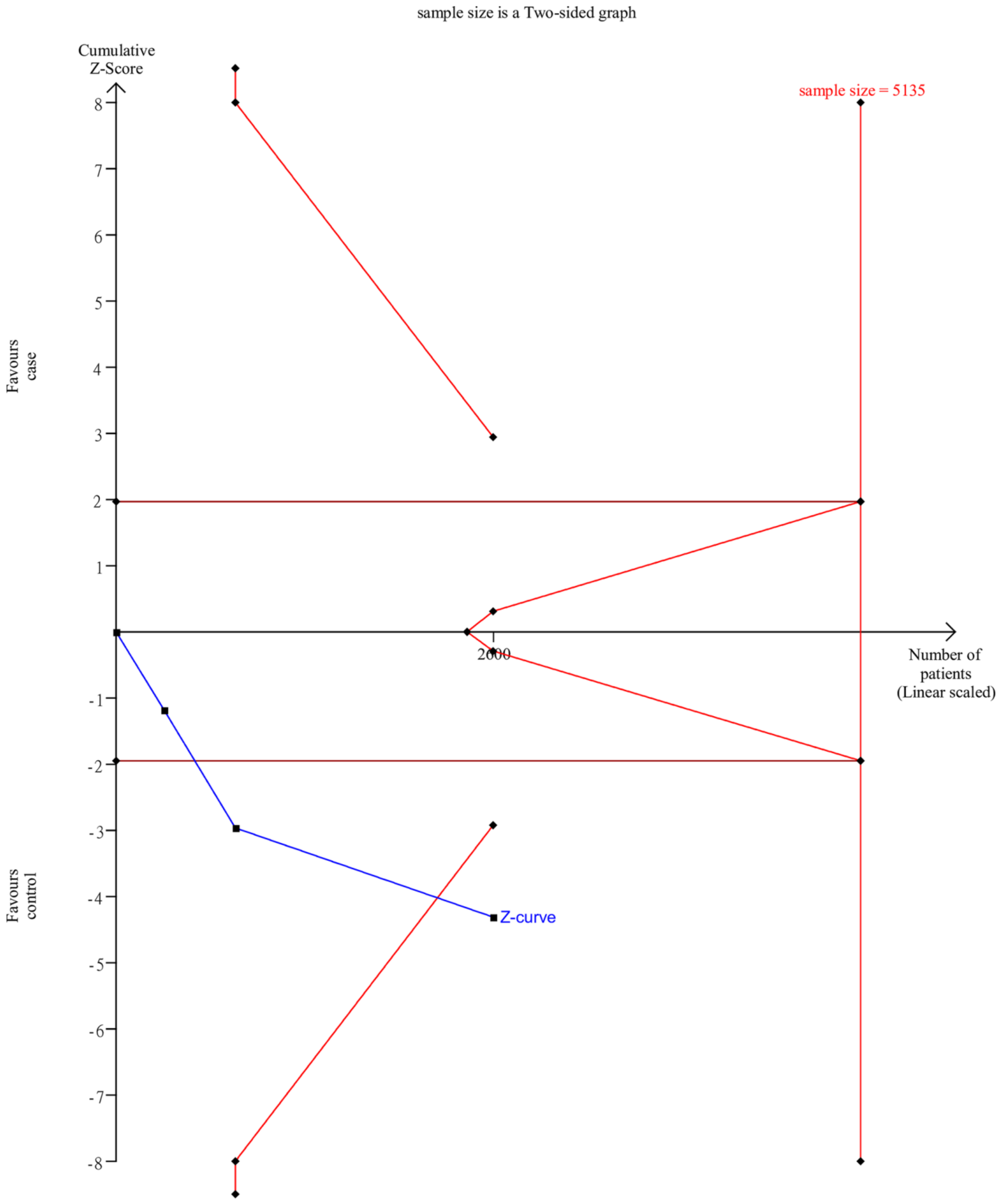

Supplementary Figure 18. TSA of the association between rs2228570 polymorphism and the risk of osteoporosis in Asians.

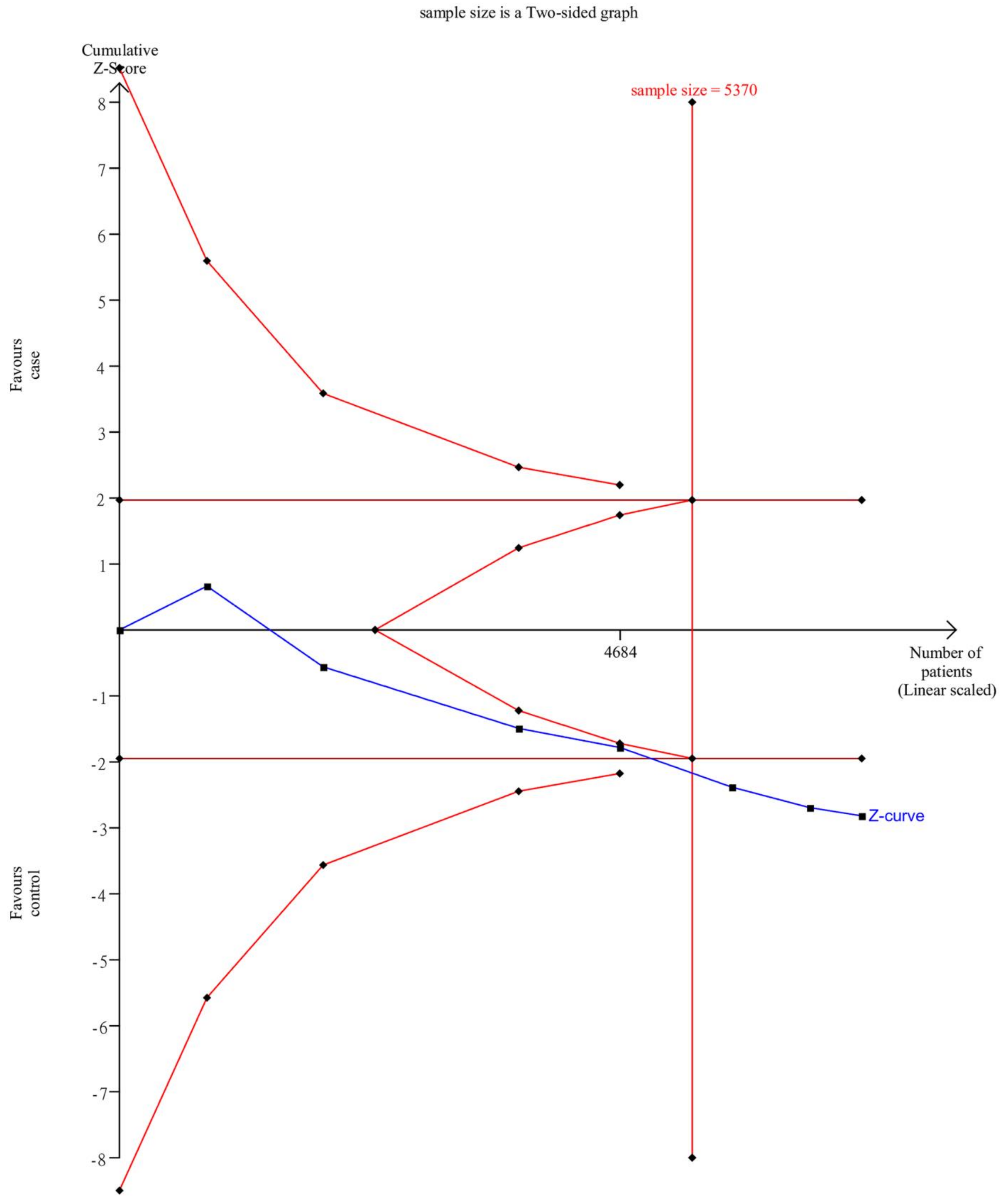

Supplementary Figure 19. TSA of the association between rs1800470 polymorphism and the risk of osteoporosis in Asians.

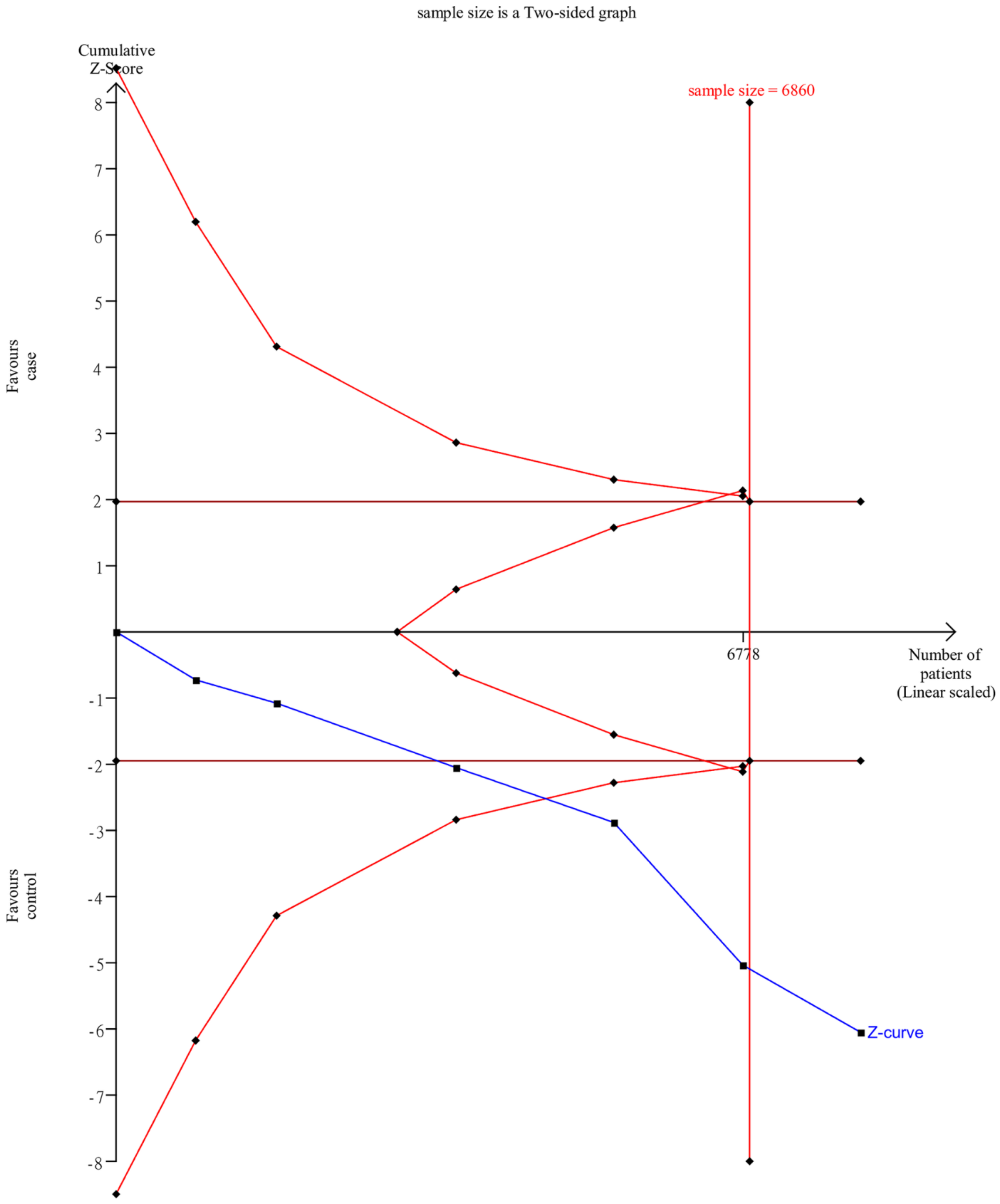

Supplementary Figure 20. TSA of the association between rs2288377 polymorphism and the risk of osteoporosis in Asians.

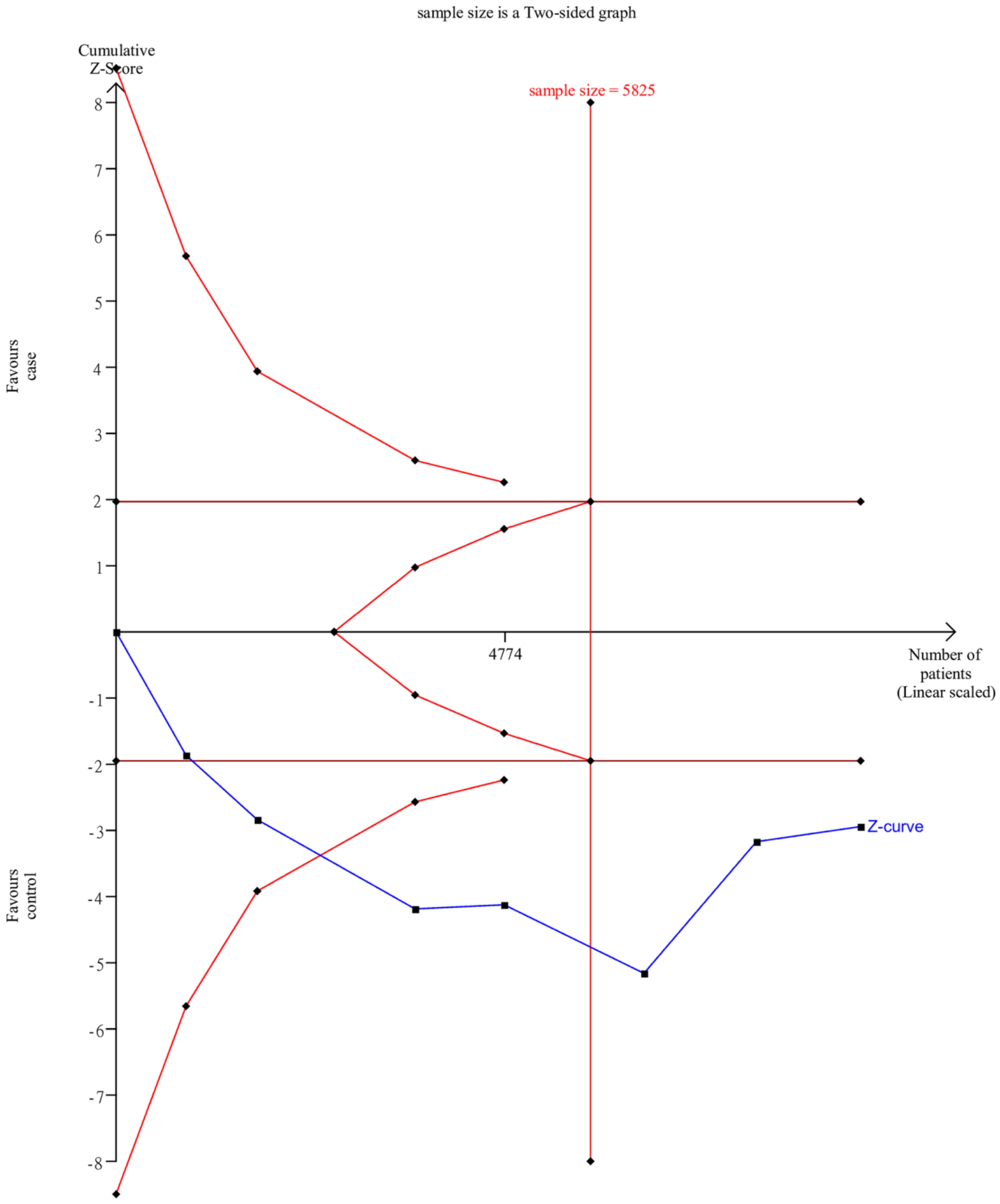

Supplementary Figure 21. TSA of the association between rs35767 polymorphism and the risk of osteoporosis in Asians.

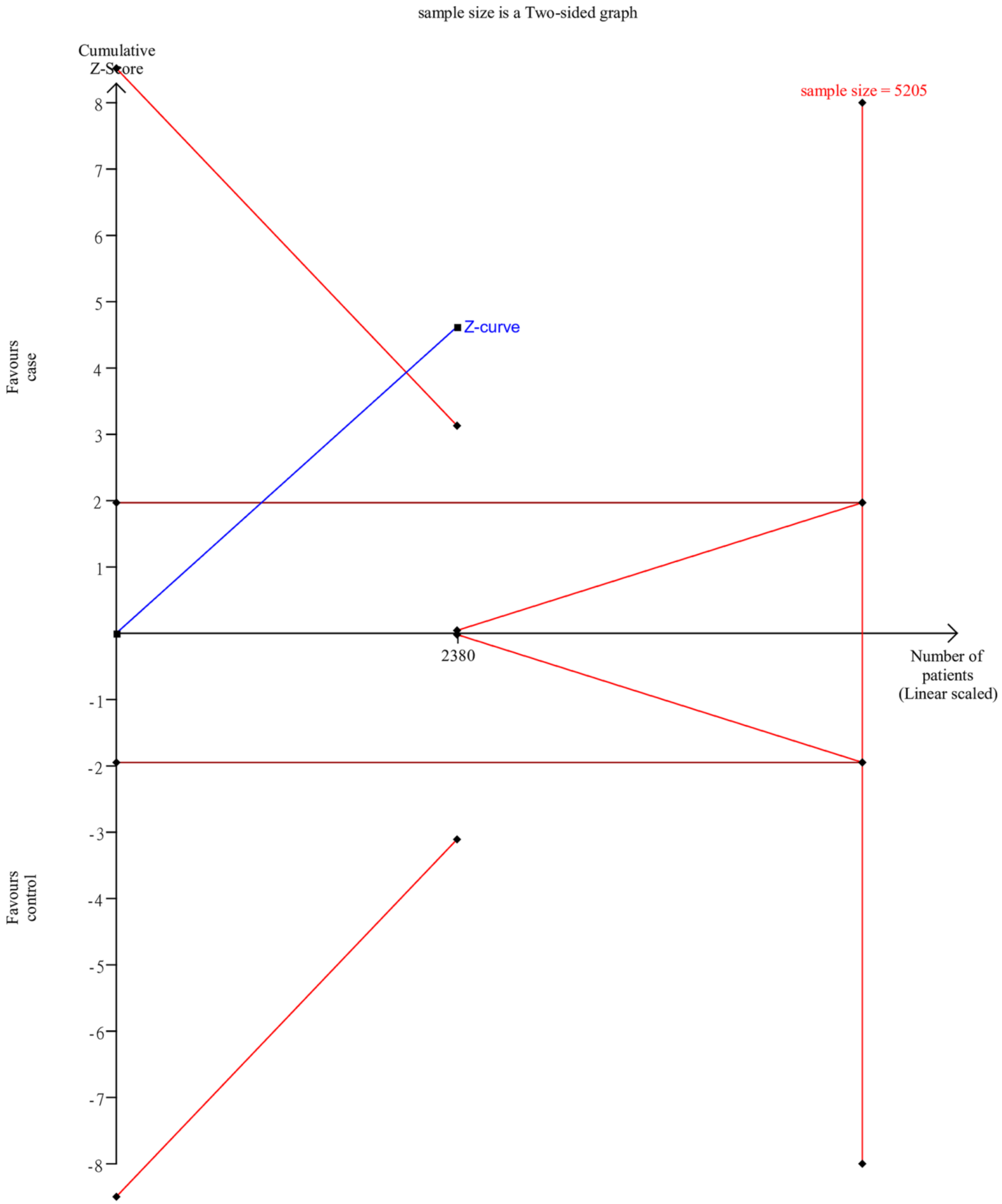

Supplementary Figure 22. TSA of the association between rs1256049 polymorphism and the risk of osteoporosis in Asians.

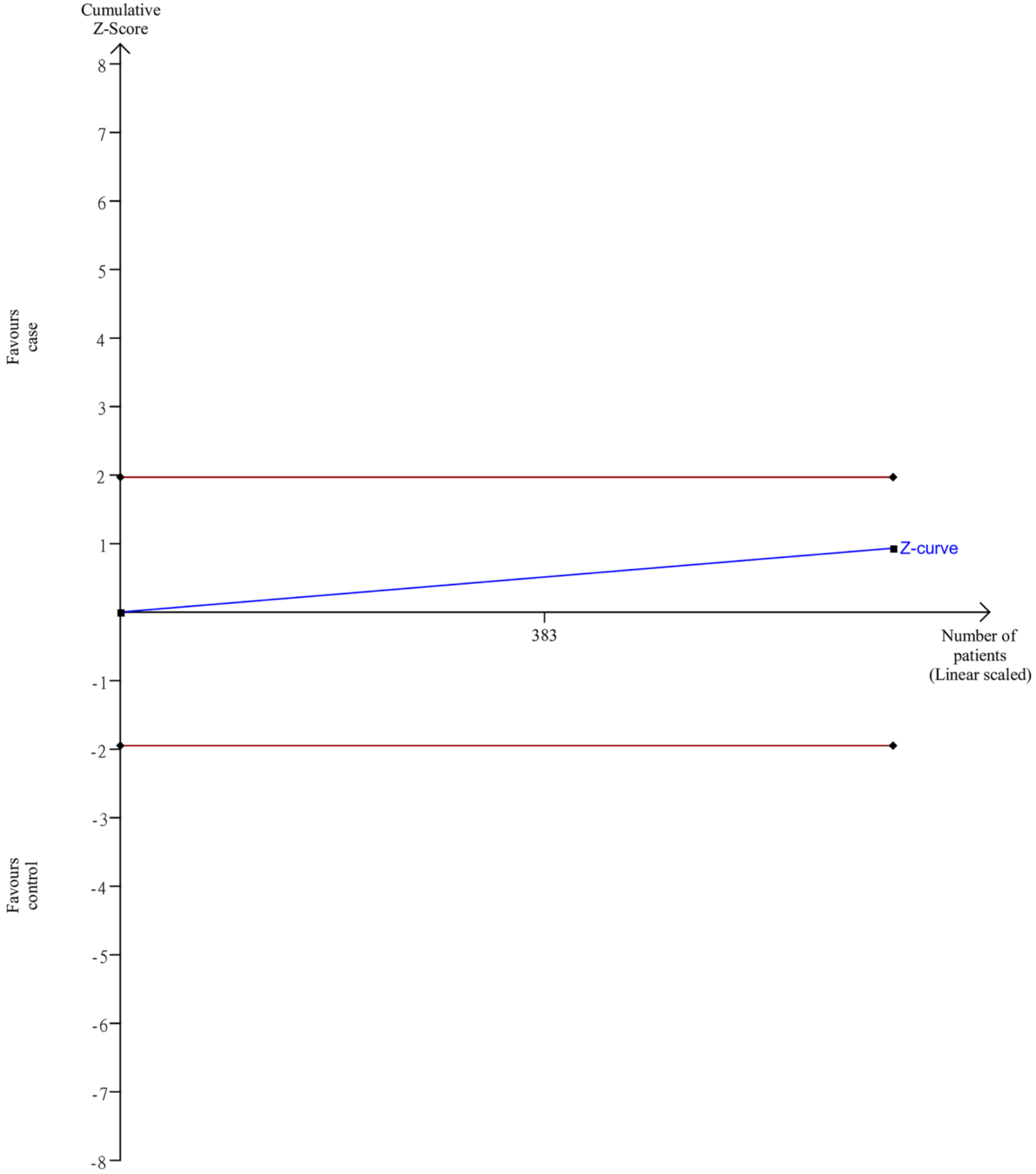

Supplementary Figure 23. TSA of the association between rs1800012 polymorphism and the risk of osteoporosis in Asians.

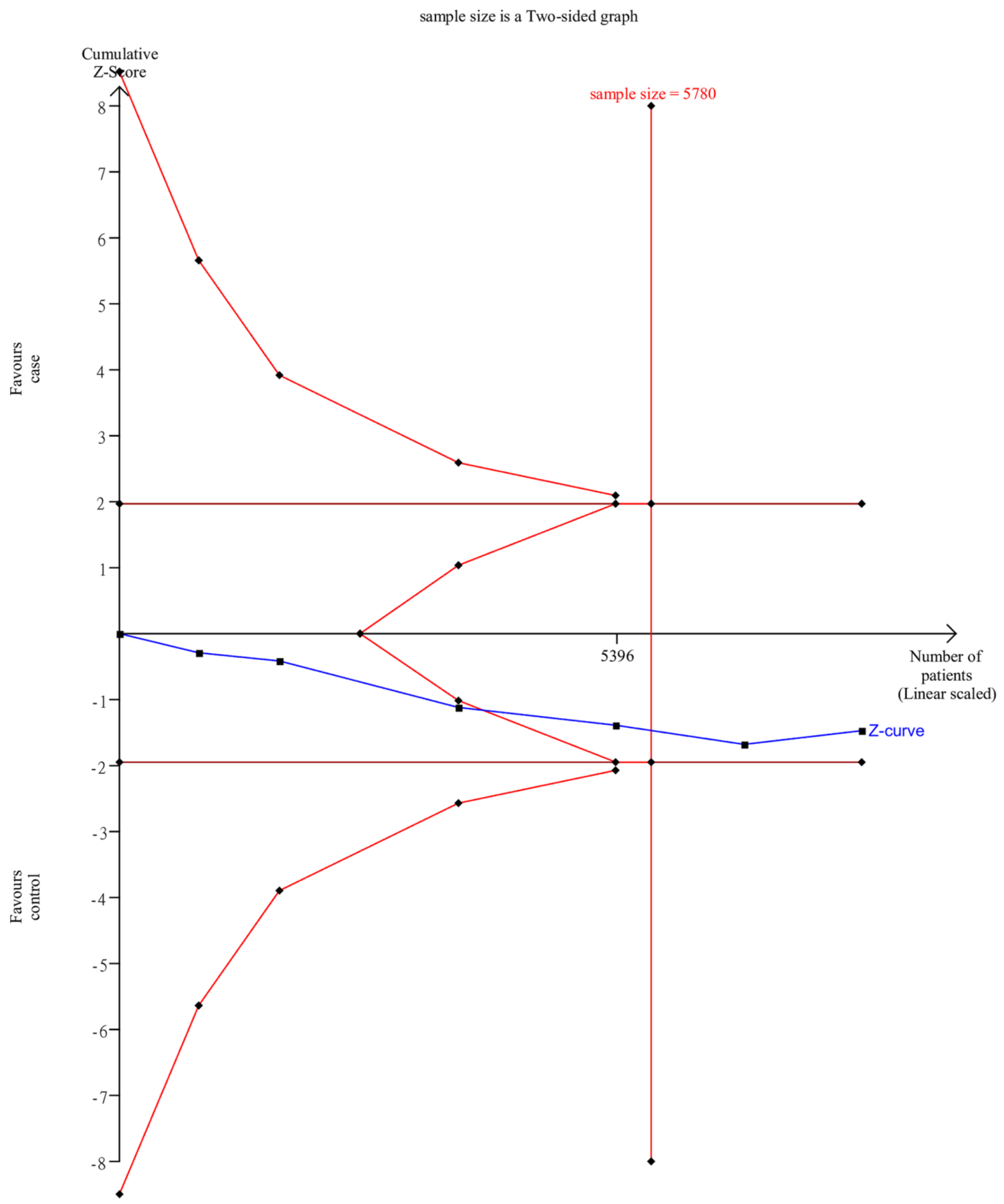

Supplementary Figure 24. TSA of the association between rs5742612 polymorphism and the risk of osteoporosis in Asians.

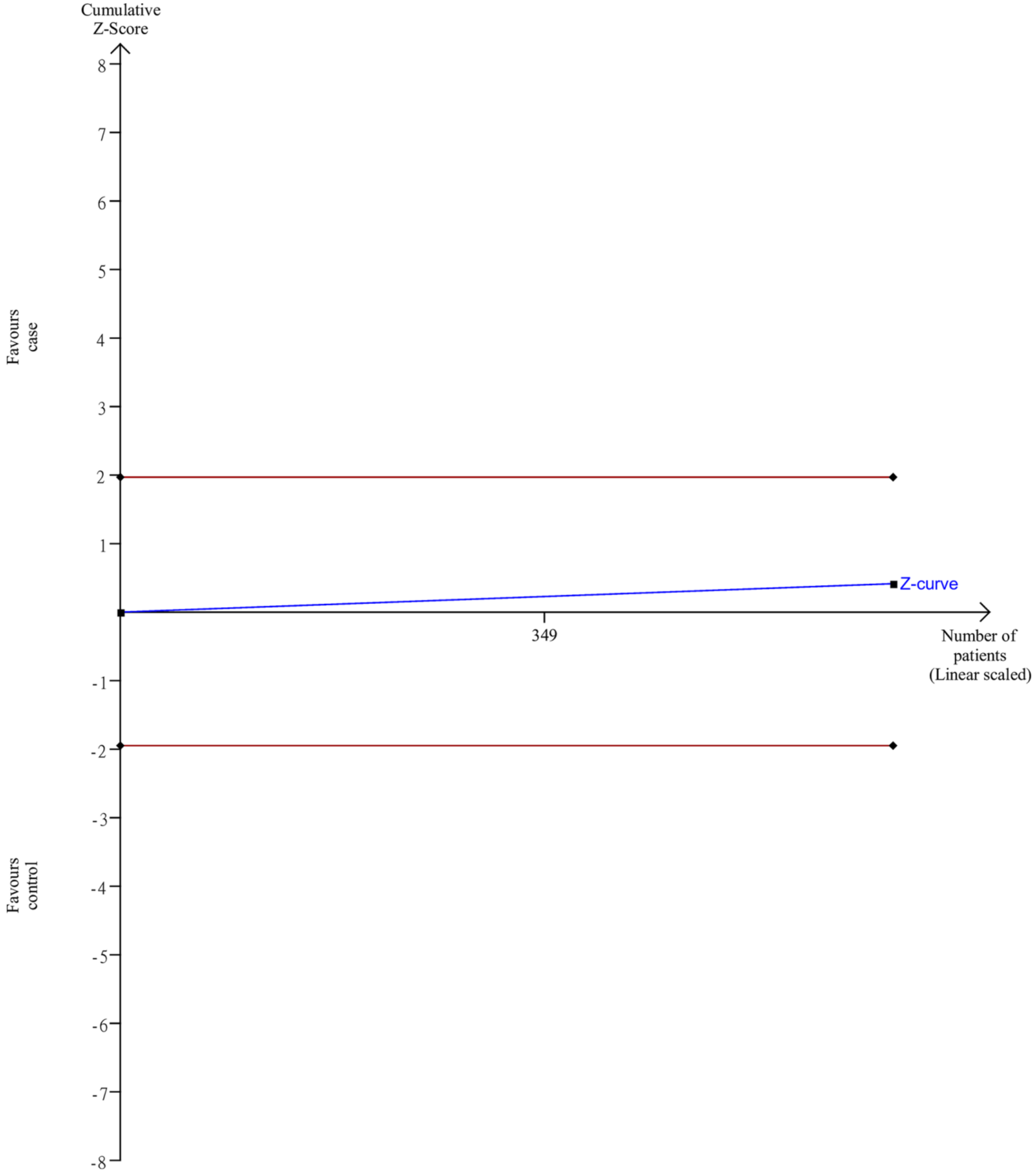

Supplementary Figure 25. TSA of the association between rs1800795 polymorphism and the risk of osteoporosis in Asians.

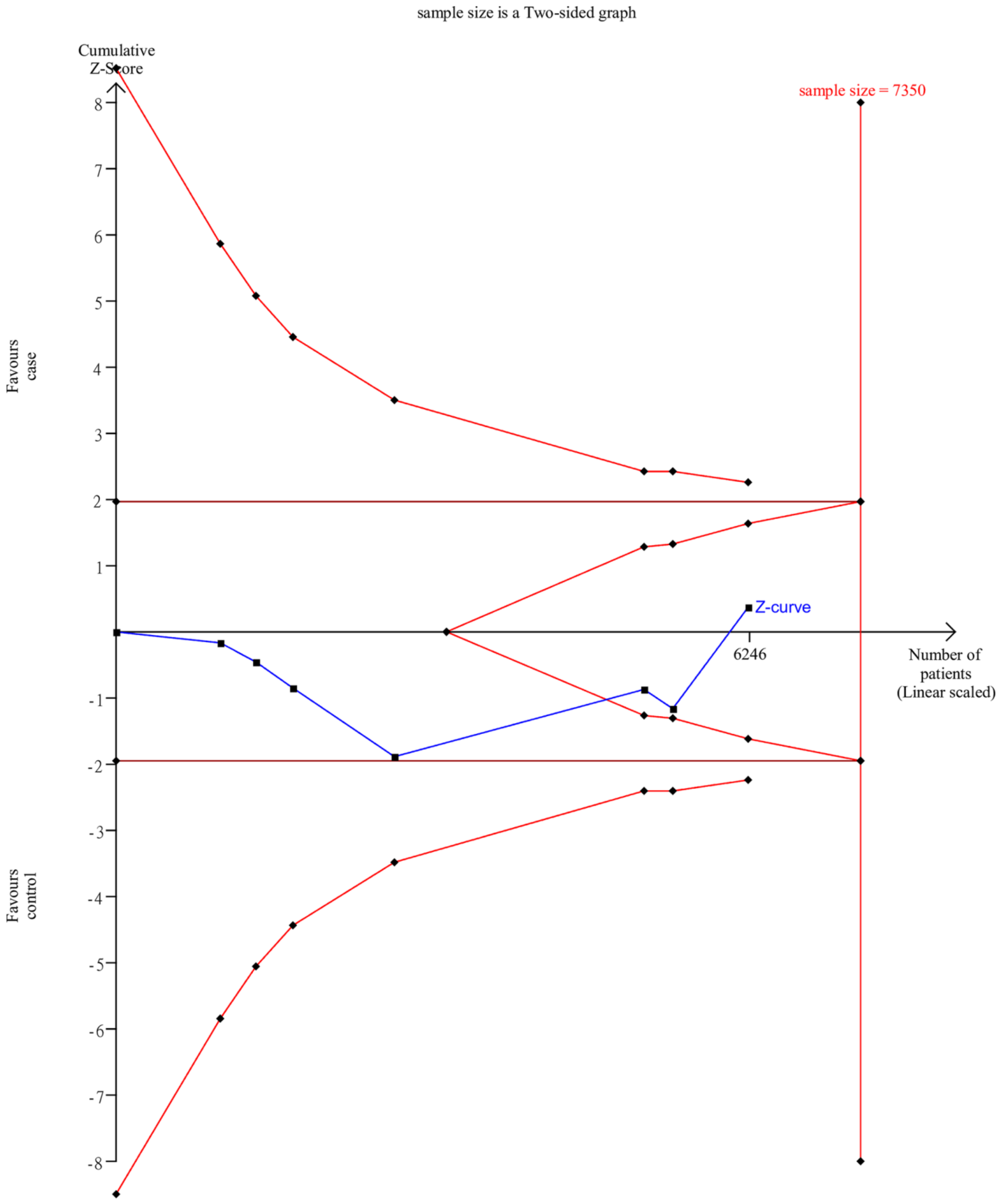

Supplementary Figure 26. TSA of the association between rs9340799 polymorphism and the risk of osteoporosis in Asians.

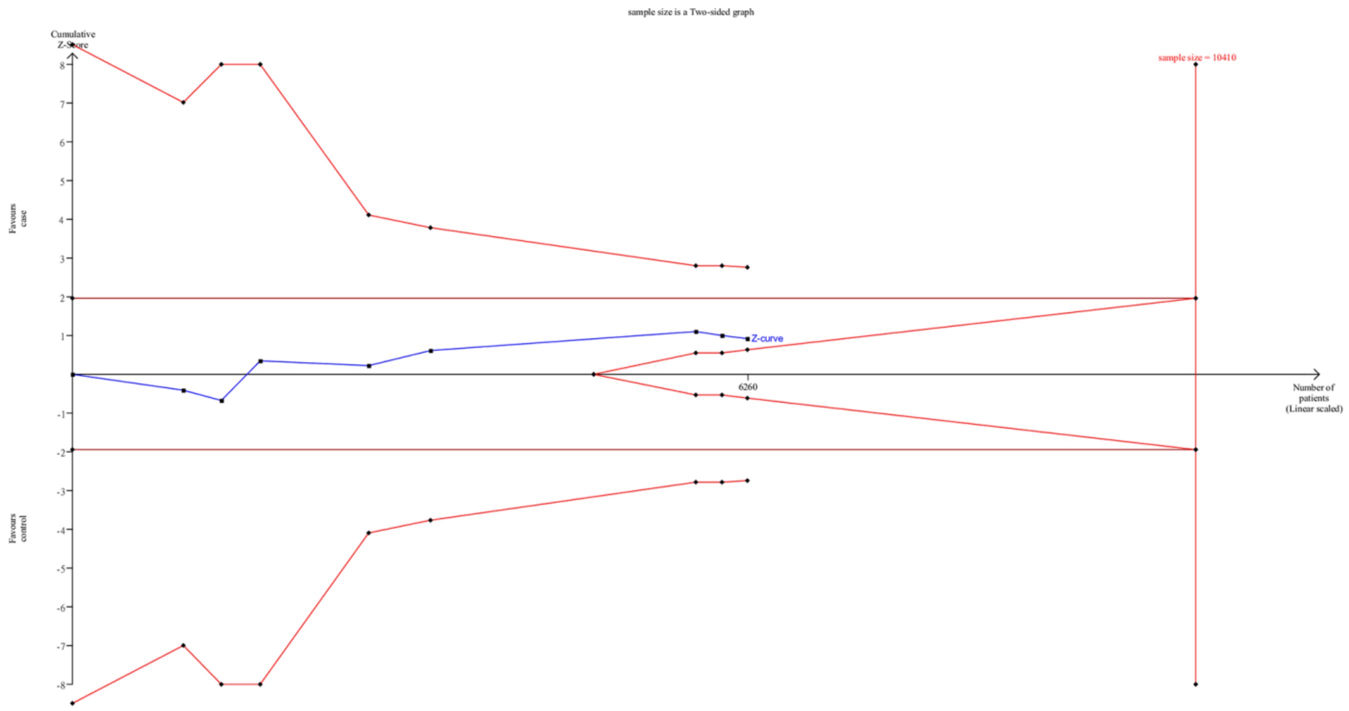

Supplementary Figure 27. TSA of the association between rs2234693 polymorphism and the risk of osteoporosis in Asians.

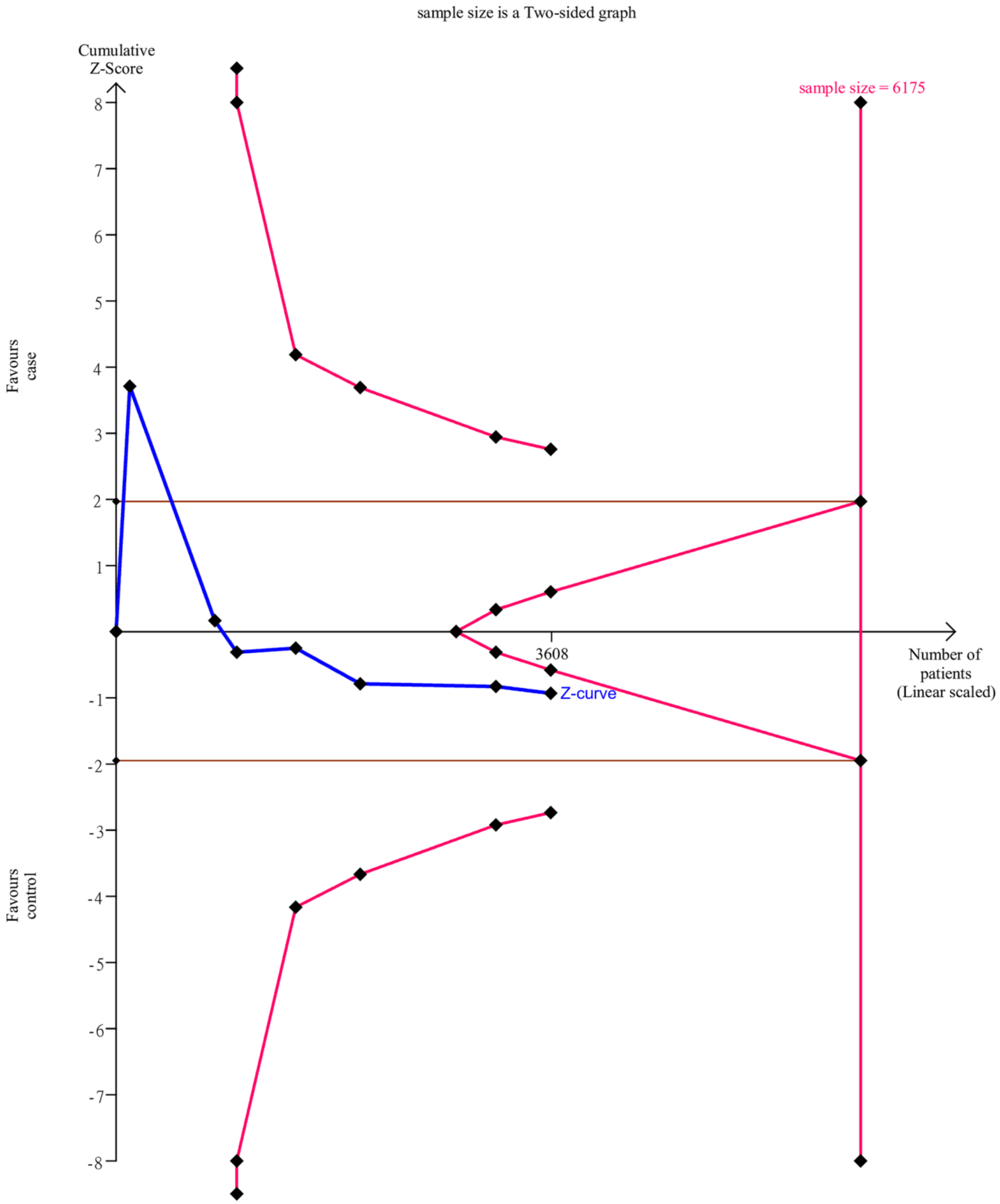

Supplementary Figure 28. TSA of the association between rs7975232 polymorphism and the risk of osteoporosis in Asians.

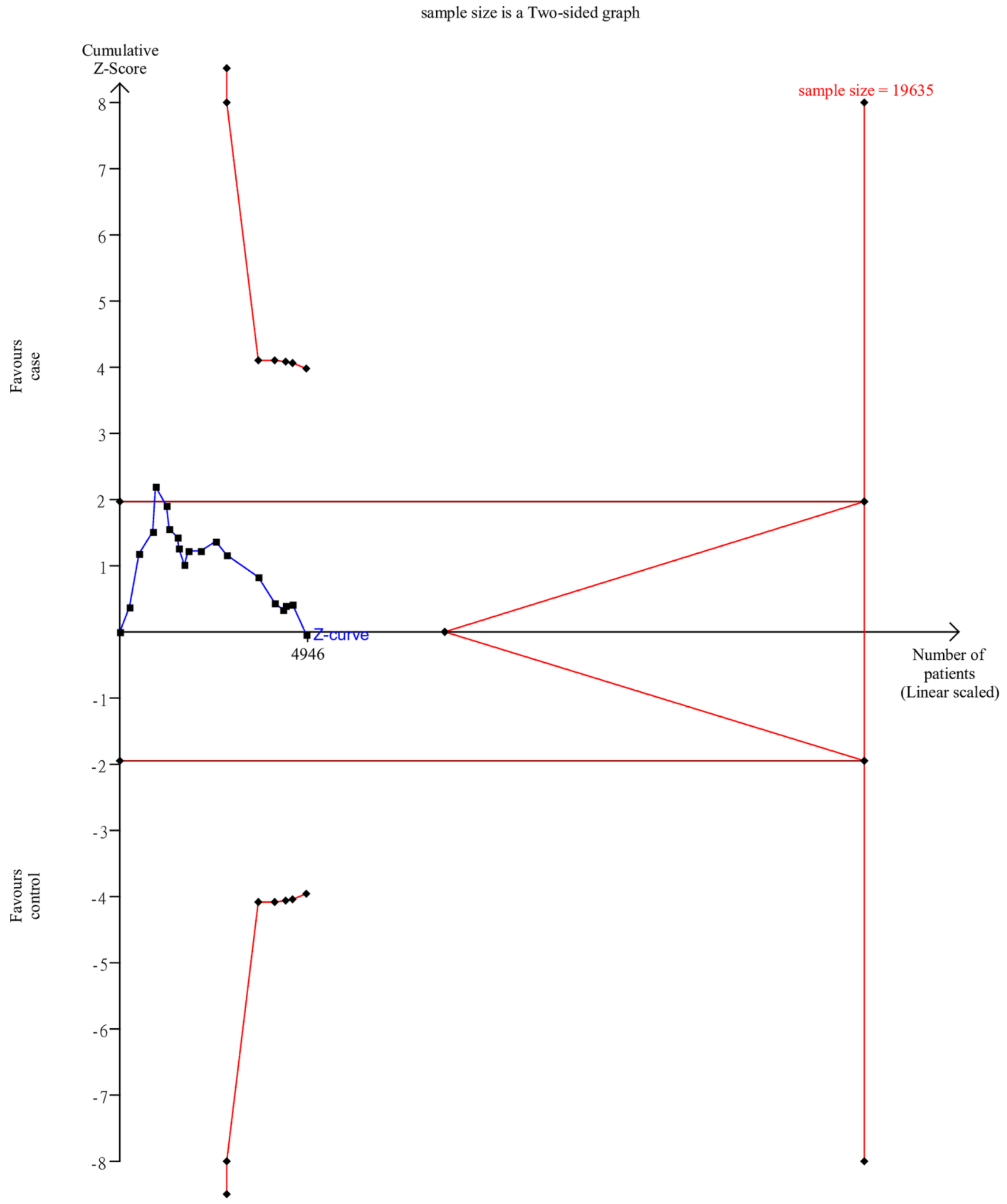

Supplementary Figure 29. TSA of the association between rs1544410 polymorphism and the risk of osteoporosis in Asians.

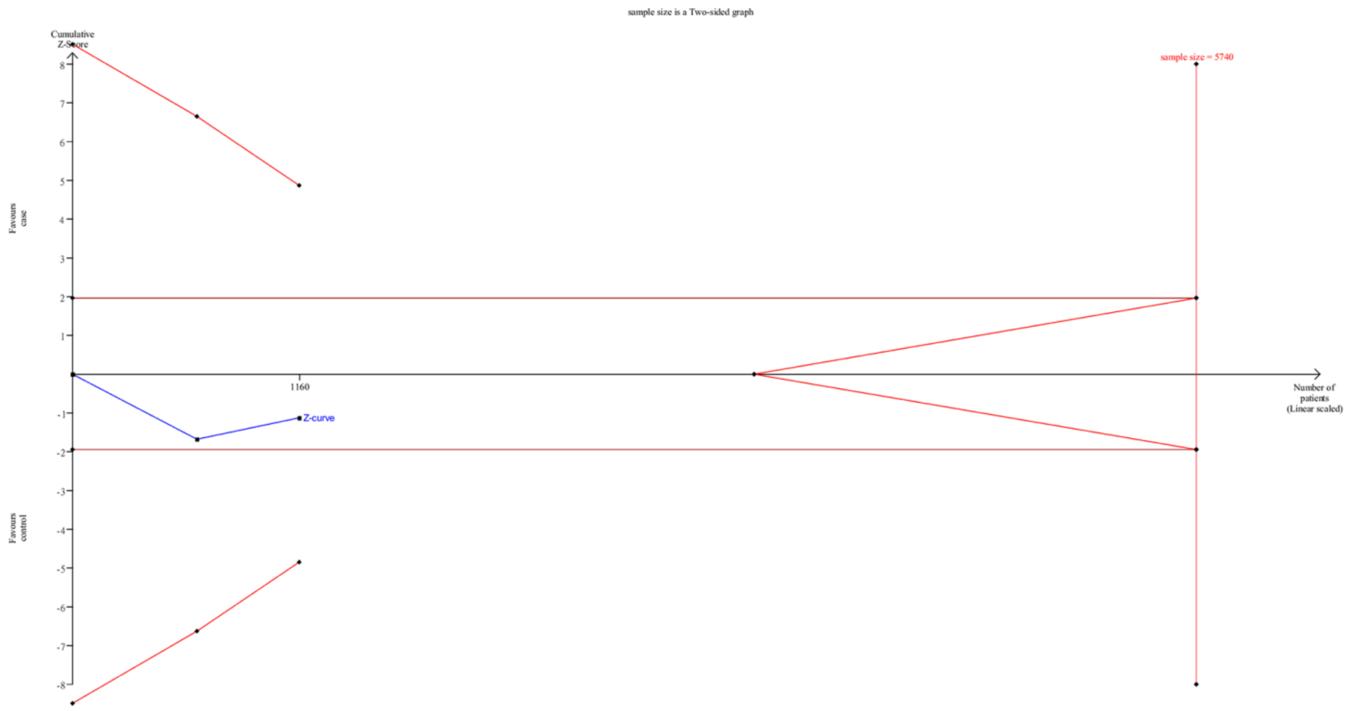

Supplementary Figure 30. TSA of the association between rs1107946 polymorphism and the risk of osteoporosis in Asians.

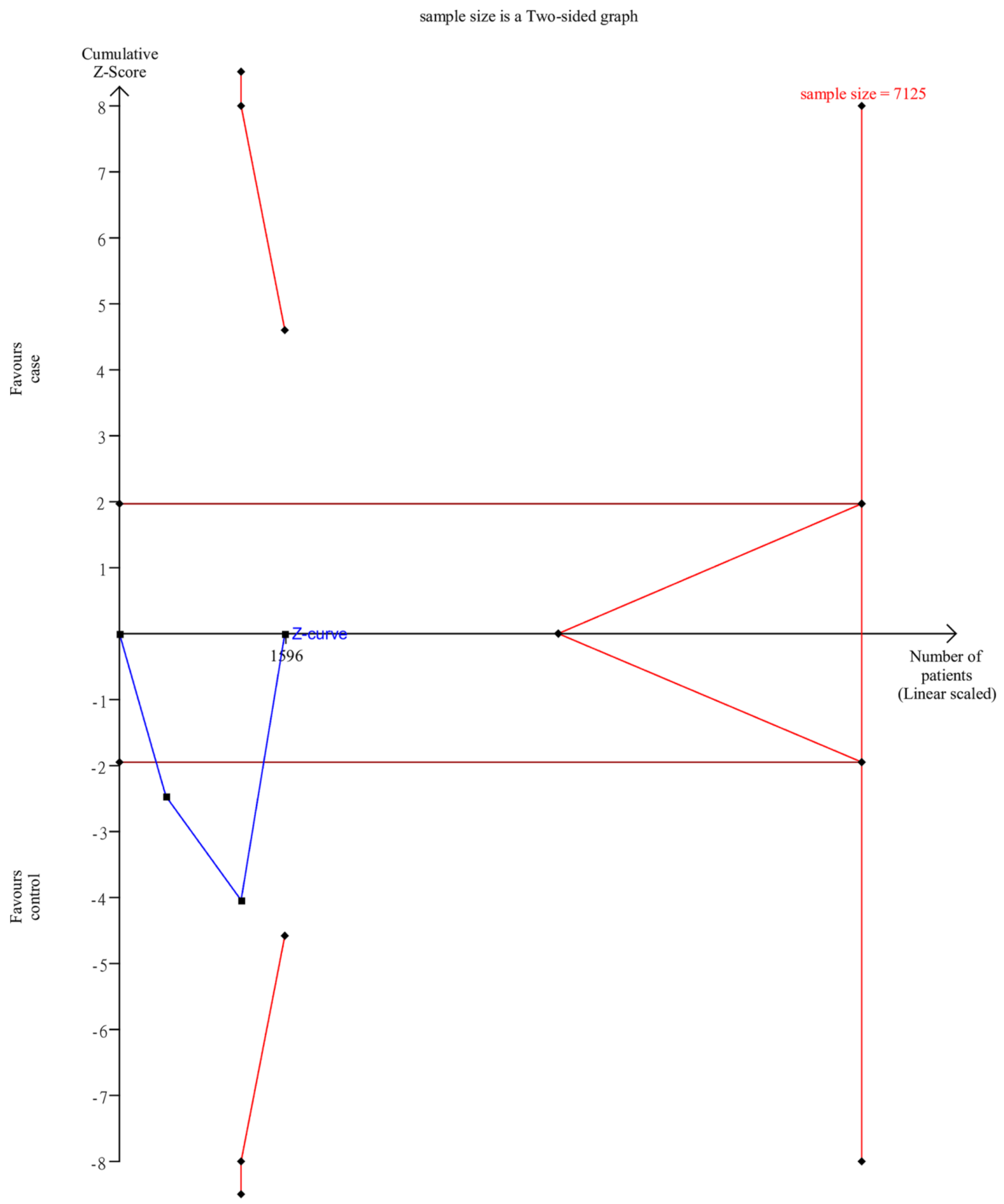

Supplementary Figure 31. TSA of the association between rs2228480 polymorphism and the risk of osteoporosis in Asians.

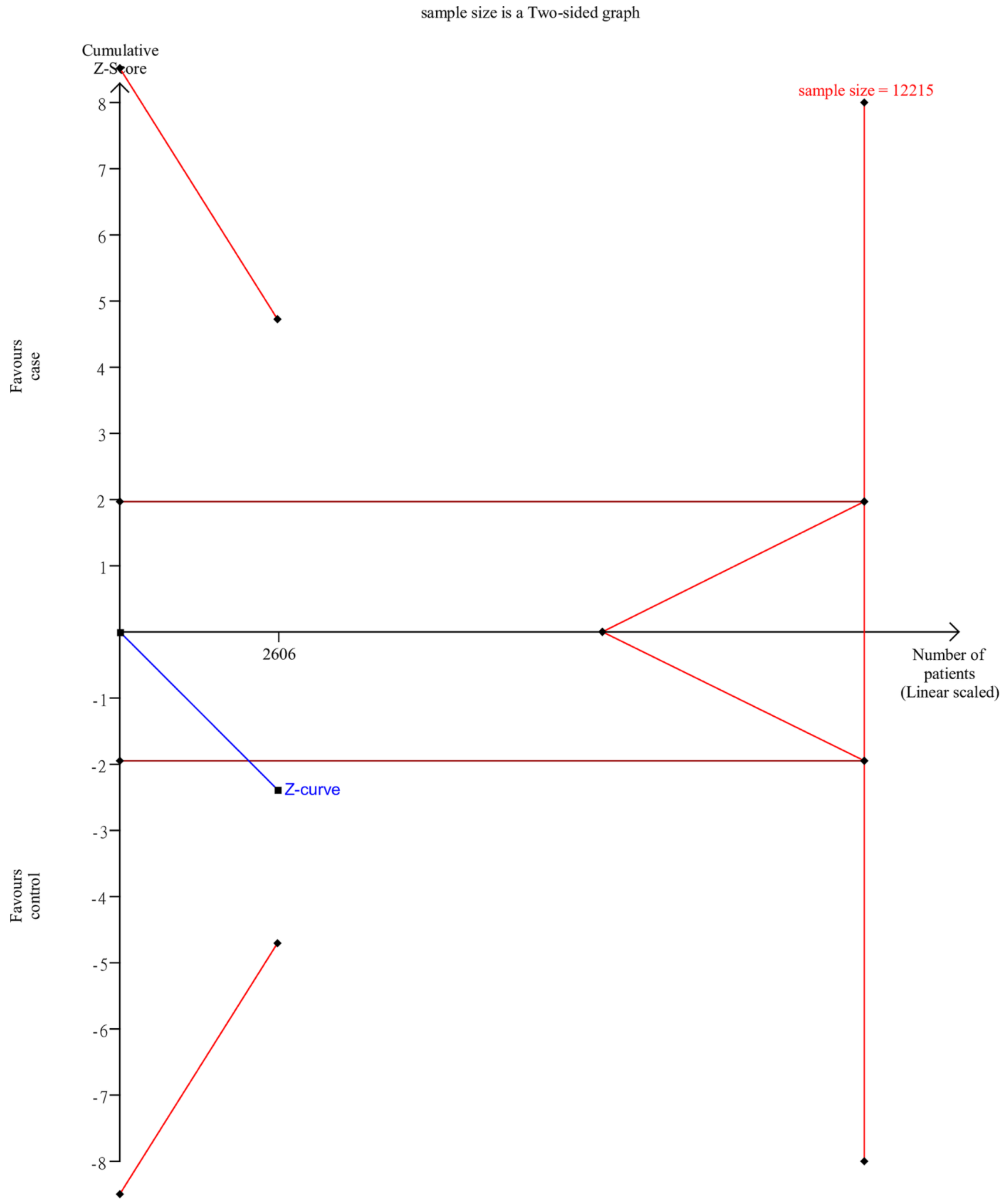

Supplementary Figure 32. TSA of the association between rs4986938 polymorphism and the risk of osteoporosis in Asians.
