# Supplementary material for: Decisive gene strategy on osteoporosis: a comprehensive whole-literature-based approach for conclusive candidate gene targets
Source: Aging (Albany NY). 2022 Apr 22;14(8):3484–528. doi: 10.18632/aging.204026 (PMC9085221; doi:10.18632/aging.204026)
Supplement: Supplementary Table 1 [file aging-14-204026-s002.pdf]

SUPPLEMENTARY TABLE

Supplementary Table 1. Search strategies.

**Relevant text of VDR ApaI**
1. genetic
2. genetic polymorphism
3. SNP
4. single nucleotide polymorphism
5. polymorphism
6. 1 or 2 or 3 or 4 or 5
**Relevant text of osteoporosis**
7. Osteoporosis
8. age- related osteoporosis
9. bone loss, age- related
10. osteoporosis, age- related
11. osteoporosis, involutional
12. osteoporosis, post-traumatic
13. osteoporosis, senile
14. senile osteoporosis
15. 7 or 8 or 9 or 10 or 11 or 12 or 13 or 14
**Relevant text of meta**
16. meta-analysis
17. meta
18. 16 or17
**Combined (Final strategy)**
19. 6 and 15 and 18

MeSH Browser: http://www.nlm.nih.gov/mesh/MBrowser.html
PubMed: http://www.ncbi.nlm.nih.gov/pubmed
Cochrane Library: http://www.thecochranelibrary.com
Embase: https://www.embase.com
